# Out-of-distribution evaluation of active learning pipelines for molecular property prediction

**DOI:** 10.1039/d5ra08055j

**Published:** 2026-01-23

**Authors:** Tianzhixi Yin, Peiyuan Gao, Gihan Panapitiya, Emily G. Saldanha

**Affiliations:** a Pacific Northwest National Laboratory USA tianzhixi.yin@gmail.com

## Abstract

Active learning (AL) has been widely applied as a strategy to reduce the data requirements of training machine learning models. Such a strategy can be especially valuable in fields where data collection is costly or time-consuming, as is the case for molecular property data. In this study, we evaluate AL for molecular property prediction, focusing on the performance on out-of-distribution (OOD) data. This OOD evaluation framework mimics the scenario found in real-world applications but is understudied in the prior literature. In our study, we focus on the prediction of solvation energy from molecular structure and develop an AL framework based on prediction uncertainties derived from Evidential Deep Learning (EDL). We started by training our model on an in-distribution training dataset and progressively augmented it with molecules from an OOD dataset sampled from PubChem, selected either randomly or using the AL strategy. We further examined generalization capabilities of AL by beginning with a subset of the in-distribution dataset, intentionally chosen to reduce initial diversity. Our results indicate that EDL demonstrates an advantage over random sampling. To further understand the behavior of the AL algorithm, we performed analysis of how the similarity between the training dataset and the held-out dataset affects the AL performance and of the distributional differences in the types of molecules selected by random sampling and AL.

## Abstract 2

Evidential deep learning–driven active learning beats random sampling for OOD solvation free-energy prediction, delivering lower test RMSE at the same labeling budget and improving generalization to unseen chemical space.

## Graphical abstract

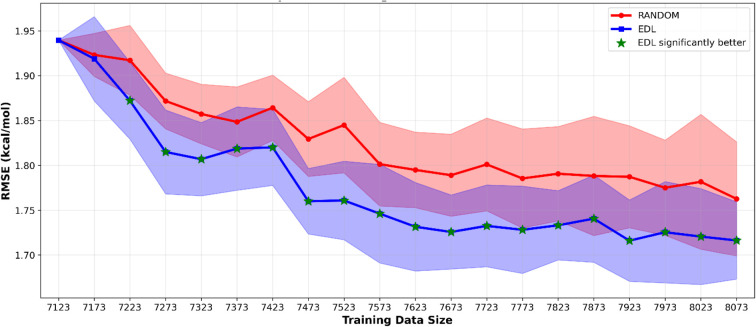

## 1. Introduction

Molecular property prediction plays a crucial role in scientific research and numerous industrial applications, such as pharmaceutical development and materials science. This task involves using computational models to predict the properties of molecules from their structure, which is essential for accelerating discovery processes and reducing experimental costs. However, one significant challenge in molecular property prediction, especially in regression tasks, is the model's ability to generalize to out-of-distribution (OOD) data–data that differ significantly from the examples seen during training. Models often struggle with OOD data, leading to reduced accuracy and reliability when applied in real-world scenarios. Active learning (AL)^1^ offers a promising solution to this problem. By employing a strategic approach to sample selection—specifically choosing instances that are likely to improve the model's performance—AL can enhance the learning process and accelerate the ability of the model to generalize to new types of molecular structures. This method focuses on querying the most informative samples, potentially enabling more robust generalizations to new, unseen datasets. Through this targeted learning approach, AL aims to build models that not only perform well on familiar data but also adapt effectively to novel molecular structures and compositions.

For the fundamentals of OOD evaluation, recent work includes both general methodology and domain-specific applications. Bibas *et al.*^2^ utilizes Single Layer Predictive Normalized Maximum Likelihood (pNML), which is shown to be effective for OOD detection across benchmarks. In the molecular domain, Ji *et al.* introduce DrugOOD,^3^ an OOD dataset curator and benchmark for AI-aided drug discovery that constructs multiple distribution shifts for drug target affinity prediction. Ghosh and co-authors' work^4^ on ensemble learning and iterative training for atom-resolved microscopy shows that model performance should be judged under experimental data distribution drifts and use uncertainty quantification and network retraining to maintain model reliability. Their later work^5^ on hypothesis-driven active learning for molecular structure–property relations emphasizes that conventional hold-out sets do not account for OOD effects in the real world, and they design an AL framework that uses Gaussian process mean functions for hypothesis learning.

However, recent literature on AL for molecular property prediction presents a mixed picture. While some studies demonstrate promising results, others find AL's performance comparable to random sampling. Amini's evidential deep learning (EDL) approach^6^ showed a clear advantage over random sampling for the QM9 dataset,^7^ and Schneider's^8^ uncertainty-based method using random forests outperformed random sampling on a subset of eMolecules.^9^ Yin's work also showed that AL has advantage over random sampling.^10^ However, Zhang's work with Bayesian semi-supervised learning^11^ across six datasets and Francoeur's study^12^ using the OPERA p*K*_a_ dataset^13^ found AL's performance indistinguishable from random sampling. Zhou's Minimum Maximum Probability Querying (MMPQ) strategy,^14^ tested on MoleculeNet,^15^ showed an advantage over random sampling and other AL baselines, though random sampling still performed strongly. A recent work^16^ on both solid-state materials and molecular design using bilinear transduction shows promise of extrapolating to OOD property predictions. These conflicting results suggest that AL's effectiveness in molecular property prediction may be context-dependent, varying with the specific dataset, property being predicted, and AL method employed.

A notable gap in literature is the limited use of truly OOD data for active learning. Typically, studies employ a hold-out dataset derived from the same data source through random data splits. In our research, we evaluate the performance of active learning on a dataset sourced from a different, more diverse origin to better understand its effectiveness in handling OOD data. This evaluation framework more closely mimics how AL would be employed in real-world applications, where the sample of new molecules to measure would be drawn from large libraries of molecular candidates. These newly sampled molecules are likely to differ from the initial training sample in substantial ways. To mimic this process, we perform a case study using the prediction of solvation energy as the target task. Solvation energy is a key parameter for molecular discovery and design across applications in chemistry, biology, pharmaceuticals, and energy storage due to its relationship with physical properties such as solubility, liquid-phase equilibrium constants, and reduction potentials. We leverage the SOMAS dataset^17^ as an initial training set and a random sample of PubChem molecules as the held-out data which the AL algorithm samples new labels from. Because the SOMAS and PubChem^18^ data were collected *via* separate mechanisms, the PubChem sample can be considered to be OOD relative to our initial SOMAS training data. This provides a robust test of the OOD performance of the AL algorithm.

## 2. Results

**Fig. 1:**
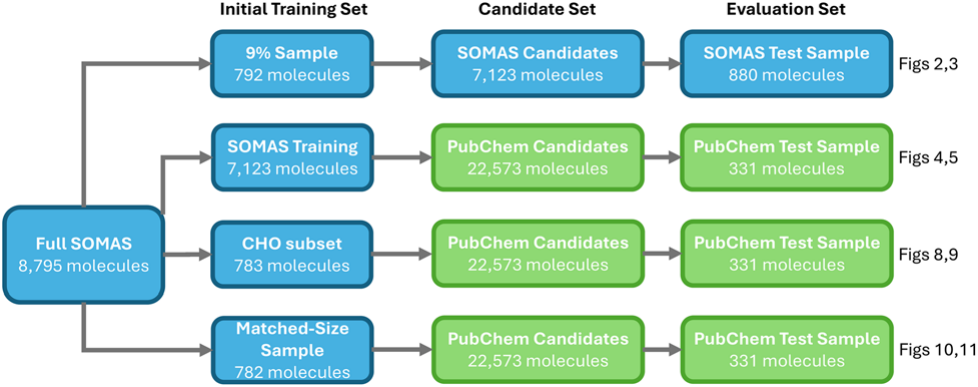
Four active-learning experiments starting from SOMAS, comparing within-SOMAS and generalization setups. Each row shows the initial training set, the candidate pool, and the evaluation set, with arrows indicating the workflow from training to candidate selection and testing.

We start with the SOMAS dataset of 8795 molecules and evaluate four active learning variants that differ in the initial training set and in-distribution *versus* OOD settings. The first is a within SOMAS run that trains on a 9% SOMAS sample, queries the remaining SOMAS candidates, and evaluates on a held out SOMAS test set. The second examines OOD generalizability by training on the SOMAS training data, selecting from PubChem candidates, and evaluating on a PubChem test set. The third uses a CHO subset of SOMAS as the seed while keeping the same PubChem candidate and test pools to probe generalizability. The fourth provides a similar size baseline by starting from a random SOMAS subset and repeating the PubChem candidate selection and evaluation, helping to understand the effects of initial training dataset on active learning. The whole evaluation framework is shown in Fig. 1.

### 2.1 Performance on SOMAS dataset

We first evaluate the AL performance on a traditional held-out dataset approach using only the SOMAS data. Initially, a small subset comprising 9% of the data was randomly selected to initialize model training, and a 9% subset was randomly sampled to be used as the test set. The remaining 80% was used as the pool of potential new molecules to sample during AL. At each iteration, we added either 50 or 500 molecules to the training set in two types of experiment, employing either EDL uncertainty-based sampling or a random selection strategy. We explored two sample sizes to compare performance in scenarios with different capacities for data collection. The performance of the deep learning model was evaluated on the test set using root mean squared error (RMSE) and *R*-squared (*R*^2^) score as the metrics. The *R*^2^ results are in the SI. We find the average result from thirty runs and compute error bars using the standard deviations of the different runs. This is also the case for the remainder of the results in the paper.

**Fig. 2:**
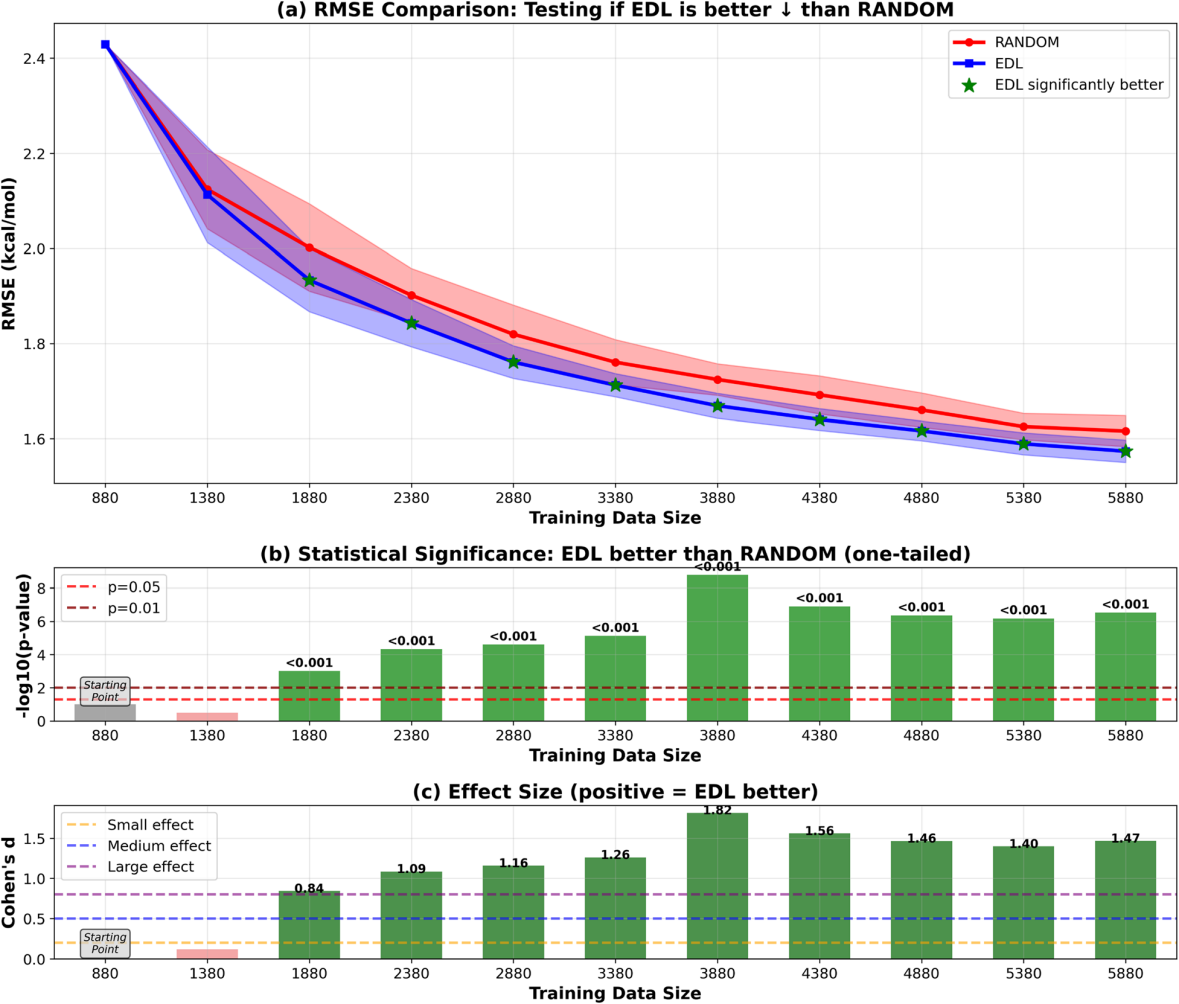
RMSE results of EDL compared with random sampling within the SOMAS dataset. Training data size is the total of the initial 9% SOMAS data and the additional held-out SOMAS samples added to the training set. Each iteration adds 500 samples to the training data. Top panel (a) shows mean ± standard deviation for RMSE, with green stars indicating statistically significant improvements (*p* < 0.05). The arrow in the panel title indicates the direction of improvement for the plotted metric. ↓ denotes that lower values indicate better performance (RMSE), whereas ↑ denotes that higher values indicate better performance (*R*^2^). Middle panel (b) displays statistical significance levels (−log_10_ *p*-values) from one-tailed tests, with horizontal dashed lines at *p* = 0.05 and *p* = 0.01 thresholds. Bottom panel (c) shows Cohen's d effect sizes, with horizontal lines indicating small (0.2), medium (0.5), and large (0.8) effect thresholds. Green coloring indicates significant improvements with meaningful effect sizes. Note: in figure labels, we use compact text forms for readability and typographic consistency: −log_10_(*p*-value) denotes −log_10_(*p*-value), and ‘*R*^2^’ denotes *R*^2^.

**Fig. 3:**
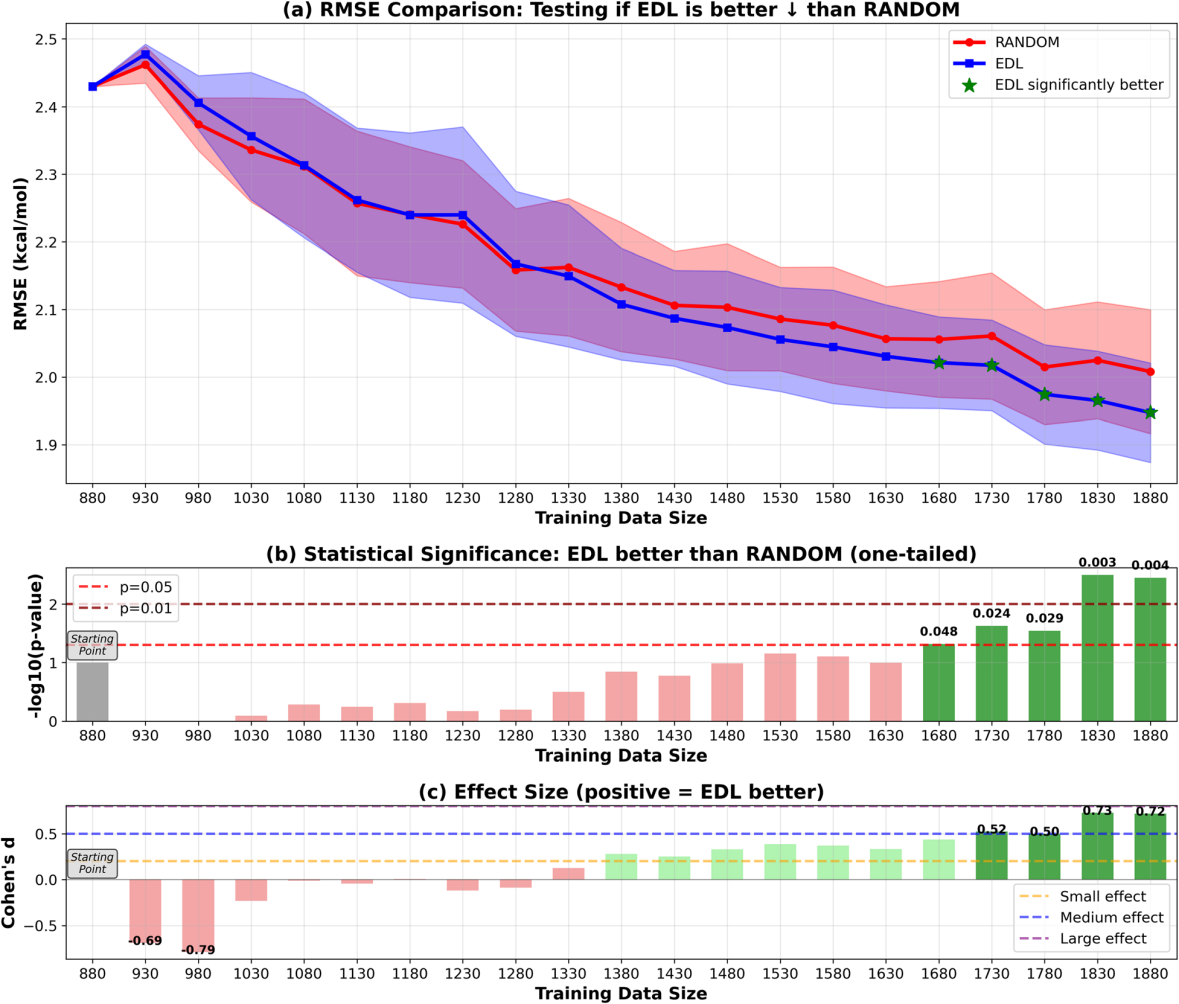
RMSE results of EDL compared with random sampling within the SOMAS dataset. Training data size is the total of the initial 9% SOMAS data and the additional held-out SOMAS samples added to the training set. Each iteration adds 50 samples. Top panel (a) shows mean ± standard deviation for RMSE, with green stars indicating statistically significant improvements (*p* < 0.05). The arrow in the panel title indicates the direction of improvement for the plotted metric. ↓ denotes that lower values indicate better performance (RMSE), whereas ↑ denotes that higher values indicate better performance (*R*^2^). Middle panel (b) displays statistical significance levels (−log_10_ *p*-values) from one-tailed tests, with horizontal dashed lines at *p* = 0.05 and *p* = 0.01 thresholds. Bottom panel (c) shows Cohen's d effect sizes, with horizontal lines indicating small (0.2), medium (0.5), and large (0.8) effect thresholds. Green coloring indicates significant improvements with meaningful effect sizes.

Our results shown in Fig. 2 and 3 indicate that EDL demonstrates advantages of around 0.05 kcal mol^−1^ in RMSE starting from the third iteration in the 500-sample experiment. In the 50-sample experiment, EDL only demonstrates advantages when reaching certain amount of training data, which is consistent with the amount of data needed to see a benefit in the 500-sample experiment.

### 2.2 Generalization to PubChem dataset

**Fig. 4:**
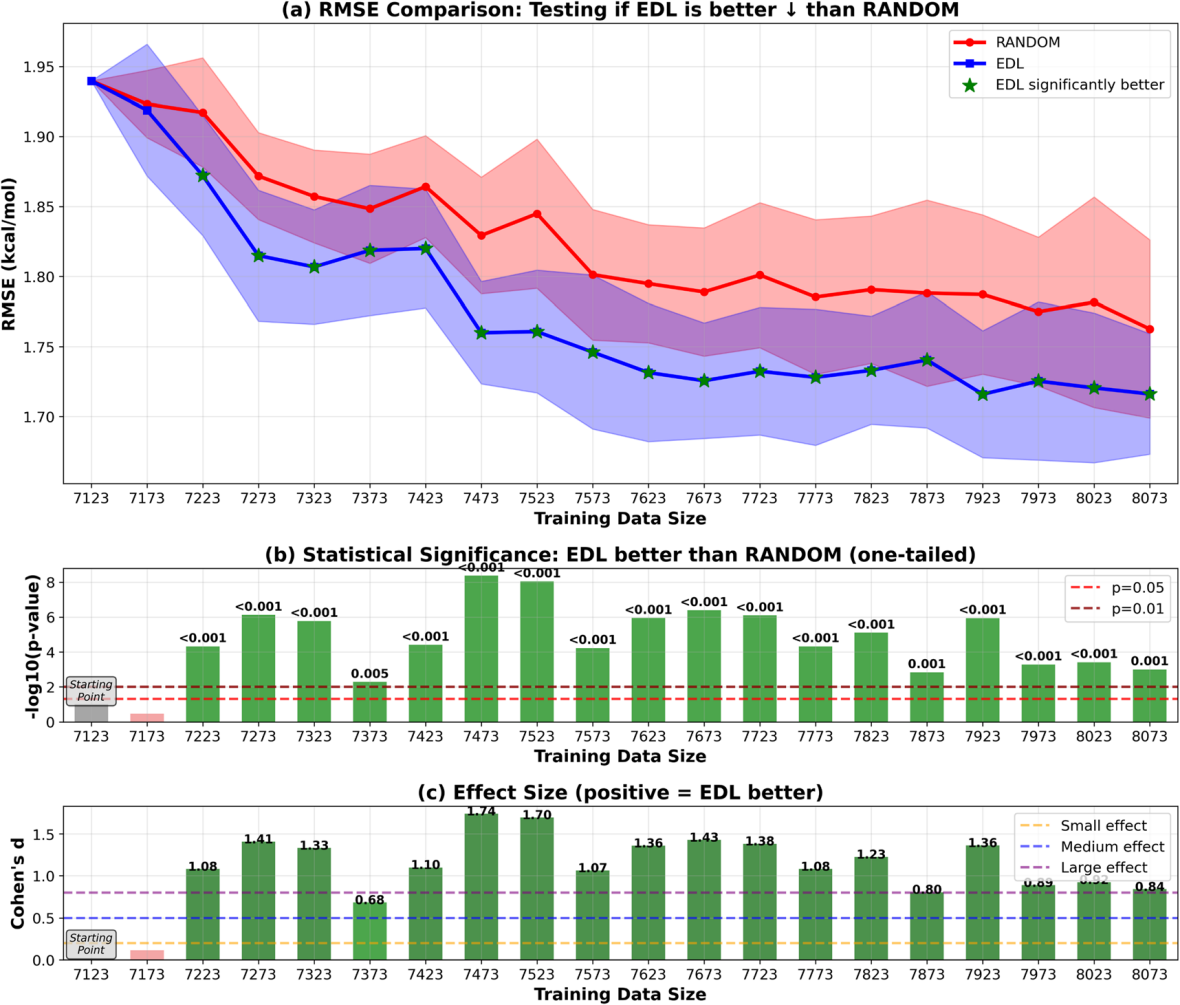
RMSE results of EDL compared with random sampling, starting with the SOMAS dataset and generalizing to the PubChem dataset with metrics reported on the PubChem test set. Training data size is the total of the SOMAS training data and the additional PubChem samples added to the training set. Each iteration adds 50 samples. Top panel (a) shows mean ± standard deviation for RMSE, with green stars indicating statistically significant improvements (*p* < 0.05). The arrow in the panel title indicates the direction of improvement for the plotted metric. ↓ denotes that lower values indicate better performance (RMSE), whereas ↑ denotes that higher values indicate better performance (*R*^2^). Middle panel (b) displays statistical significance levels (−log_10_ *p*-values) from one-tailed tests, with horizontal dashed lines at *p* = 0.05 and *p* = 0.01 thresholds. Bottom panel (c) shows Cohen's d effect sizes, with horizontal lines indicating small (0.2), medium (0.5), and large (0.8) effect thresholds. Green coloring indicates significant improvements with meaningful effect sizes.

**Fig. 5:**
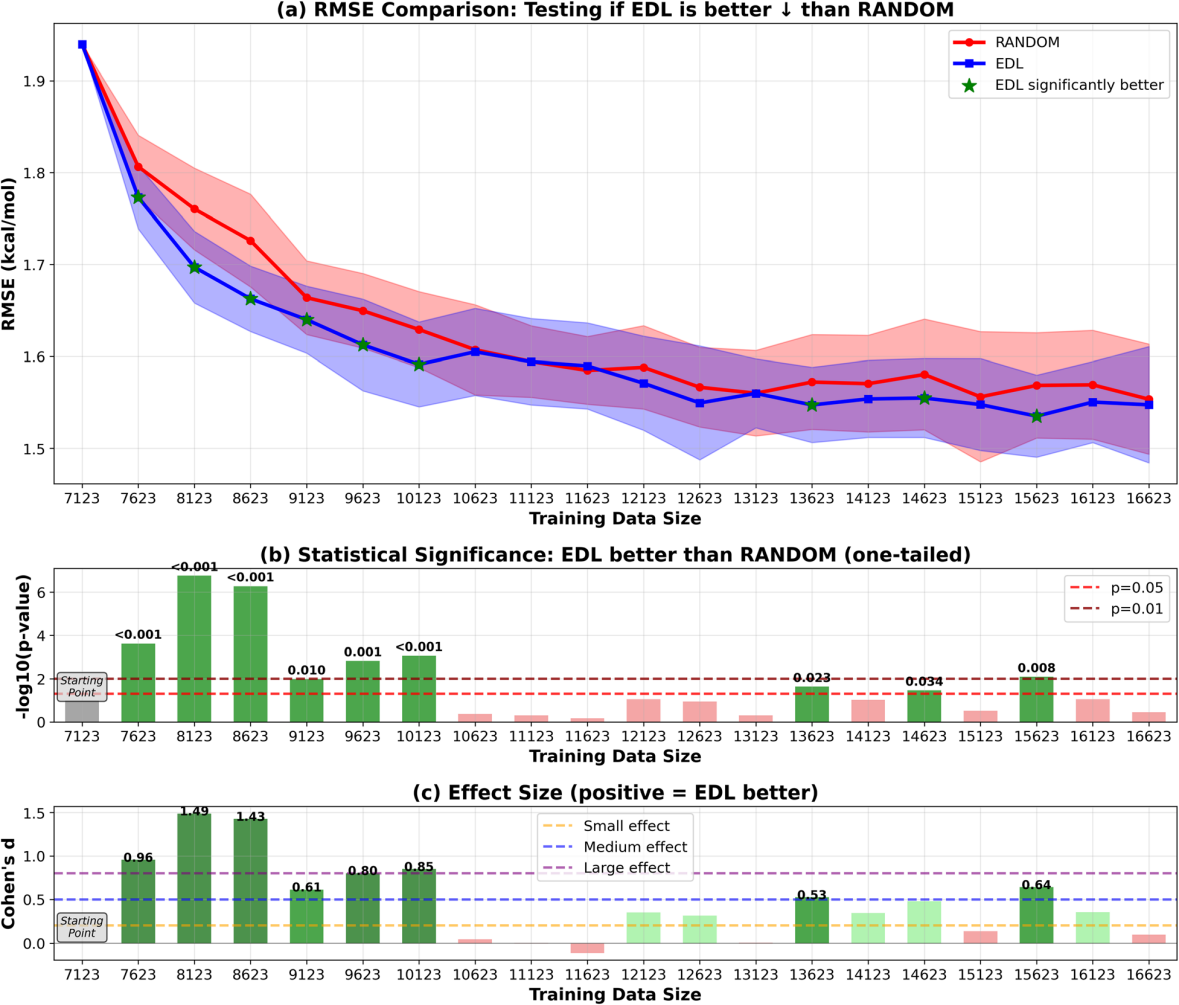
RMSE results of EDL compared with random sampling, starting with the SOMAS dataset and generalizing to the PubChem dataset. Training data size is the total of the SOMAS training data and the additional PubChem samples added to the training set. Each iteration adds 500 samples. Top panel (a) shows mean ± standard deviation for RMSE, with green stars indicating statistically significant improvements (*p* < 0.05). The arrow in the panel title indicates the direction of improvement for the plotted metric. ↓ denotes that lower values indicate better performance (RMSE), whereas ↑ denotes that higher values indicate better performance (*R*^2^). Middle panel (b) displays statistical significance levels (−log_10_ *p*-values) from one-tailed tests, with horizontal dashed lines at *p* = 0.05 and *p* = 0.01 thresholds. Bottom panel (c) shows Cohen's d effect sizes, with horizontal lines indicating small (0.2), medium (0.5), and large (0.8) effect thresholds. Green coloring indicates significant improvements with meaningful effect sizes.

To evaluate the generalization capability of the AL approach to the PubChem dataset, we initially train the model on the entire SOMAS dataset. After performing initial training on the SOMAS data, we then incrementally add molecules from the PubChem candidate dataset, which contains 22 573 molecules, 50 or 500 at a time, and assess the model's performance on a held-out PubChem test set that contains 331 molecules. This process is again conducted using both EDL and a random selection strategy. The results are shown in Fig. 4 and 5. Our findings indicate that EDL exhibits a distinct advantage as more molecules are incorporated in the 50-sample experiment. Specifically, as we approach the end of the active learning process, both methods show signs of convergence with a plateau in the RMSE. However, EDL maintains an approximate advantage of 0.05 kcal mol^−1^ in RMSE compared to the random selection strategy at the convergence, underscoring its efficacy in leveraging the additional data for improved model performance. The model trained with AL is able to exceed the final performance of the random sampling model with only 37% of the number of data points sampled from the PubChem data. For the 500-sample experiment, we see that EDL only demonstrates advantages in the early iterations, as more training data are available, random sampling catches up with EDL.

**Fig. 6:**
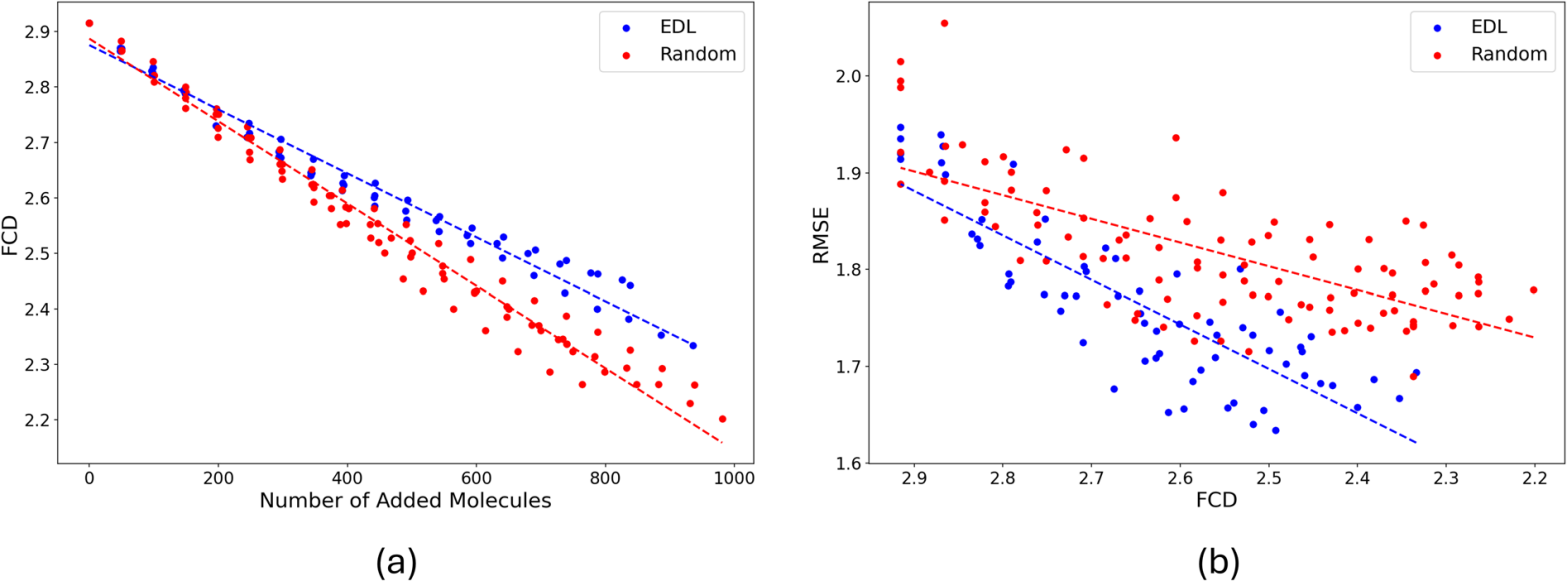
(a) FCD between training set and PubChem pool set by EDL and random sampling according to number of molecules added to the training set. (b) RMSE on the PubChem testing set *vs.* FCD between training set and PubChem pool set by EDL and random sampling.

We investigated the similarity between the training set and the hold-out set using the Fréchet ChemNet Distance (FCD).^19^ Our analysis reveals that as active learning progresses, the similarity between these sets increases as shown in Fig. 6(a). This improvement in similarity corresponds with enhanced performance on the PubChem test set, evidenced by a decrease in RMSE, shown in Fig. 6(b). In comparing AL and random sampling methodologies, we observe that random sampling approaches the PubChem hold-out set distribution faster. However, AL demonstrates superior performance on the test set, despite exhibiting greater divergence from the overall hold-out set distribution. This suggests that AL may be more effective at identifying informative samples for model improvement, even if the resulting sample distribution is less representative of the broader dataset.

As reported in Fig. 6, the distributional shift between the in-domain SOMAS dataset and the OOD PubChem dataset is between 2.2 to 2.9. For context, we also estimate an in-domain baseline by computing an internal SOMAS to SOMAS FCD using bootstrap resampling (20 iterations, half-sample size), obtaining a mean FCD of 0.445 with standard deviation 0.027. The larger SOMAS to PubChem FCD compared to the SOMAS internal baseline indicates a chemical distribution shift.

**Fig. 7:**
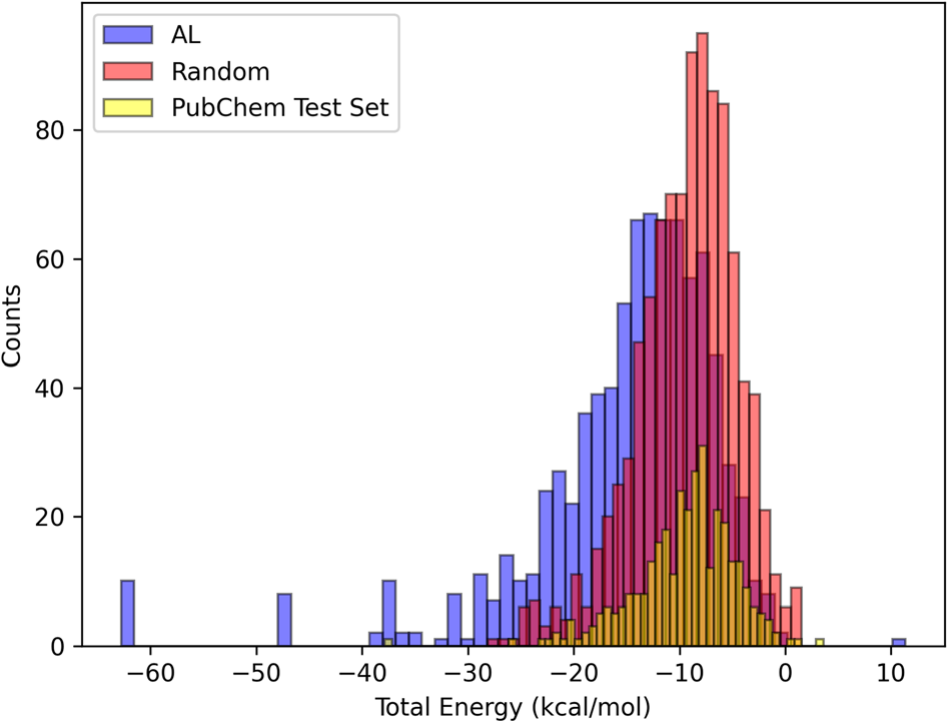
Histograms of the distributions of the total energy of the molecules selected by EDL and random sampling, also plotted is the distribution of the PubChem test set. The *y* axis indicates counts (number of example molecules) per category.

We also compared the solvation energy distributions of the molecules selected by AL and random sampling. Our findings in Fig. 7 indicate that AL is able to select molecules that are outliers, resulting in a distribution with a long left tail. In contrast, random sampling primarily selects molecules with the solvation energies in the most likely range, leading to a more concentrated distribution around the mean. Here we see that random sampling produces a distribution that is more similar to the distribution of the PubChem test set.

### 2.3 Analysis of subset generalization

**Fig. 8:**
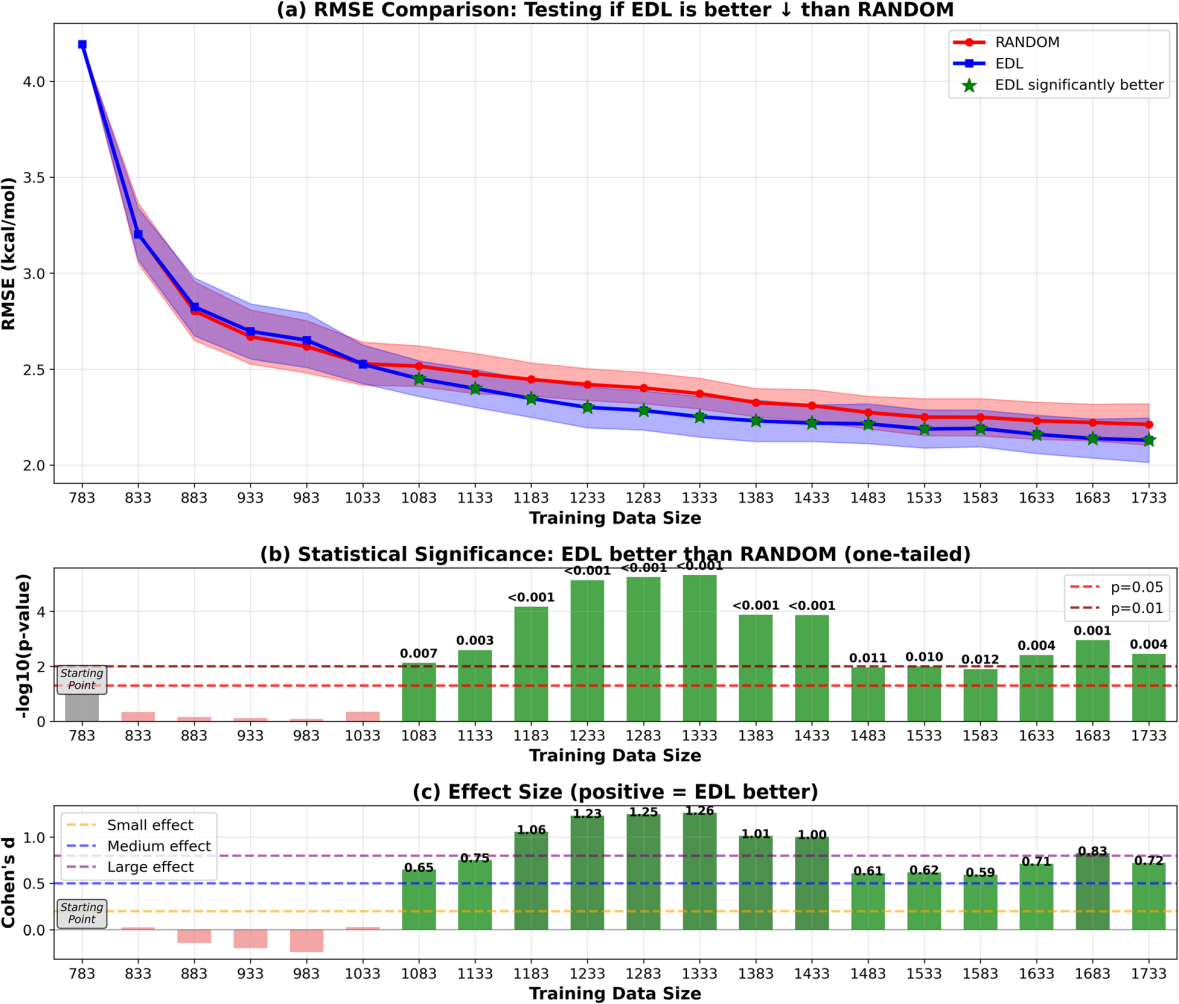
RMSE results of EDL compared with random sampling, starting with the CHO subset from SOMAS and generalizing to the PubChem dataset. Each iteration adds 50 samples. Top panel (a) shows mean ± standard deviation for RMSE, with green stars indicating statistically significant improvements (*p* < 0.05). The arrow in the panel title indicates the direction of improvement for the plotted metric. ↓ denotes that lower values indicate better performance (RMSE), whereas ↑ denotes that higher values indicate better performance (*R*^2^). Middle panel (b) displays statistical significance levels (−log_10_ *p*-values) from one-tailed tests, with horizontal dashed lines at *p* = 0.05 and *p* = 0.01 thresholds. Bottom panel (c) shows Cohen's d effect sizes, with horizontal lines indicating small (0.2), medium (0.5), and large (0.8) effect thresholds. Green coloring indicates significant improvements with meaningful effect sizes.

In addition to initiating the model training with the entire SOMAS dataset, we also explored starting from a specifically chosen subset of SOMAS. This subset was selected to intentionally reduce the initial diversity of the training dataset, allowing us to evaluate the active learning framework's capability to generalize to a more diverse dataset as compared to random sampling. In this subset which we call CHO, only the molecules that include only C, H and O elements were selected. The RMSE performance result is shown in Fig. 8 for the 50-sample experiment. We see that the initial performance of the model is much worse than starting with the full SOMAS training data with an RMSE around 4.2 kcal mol^−1^ and an *R*^2^ around 0.2. Both random sampling and AL lead to a rapid improvement in performance during the initial iterations, with EDL demonstrating a slight advantage over random sampling after approximately seven iterations after which AL maintains a 0.05 kcal mol^−1^ advantage in RMSE.

**Fig. 9:**
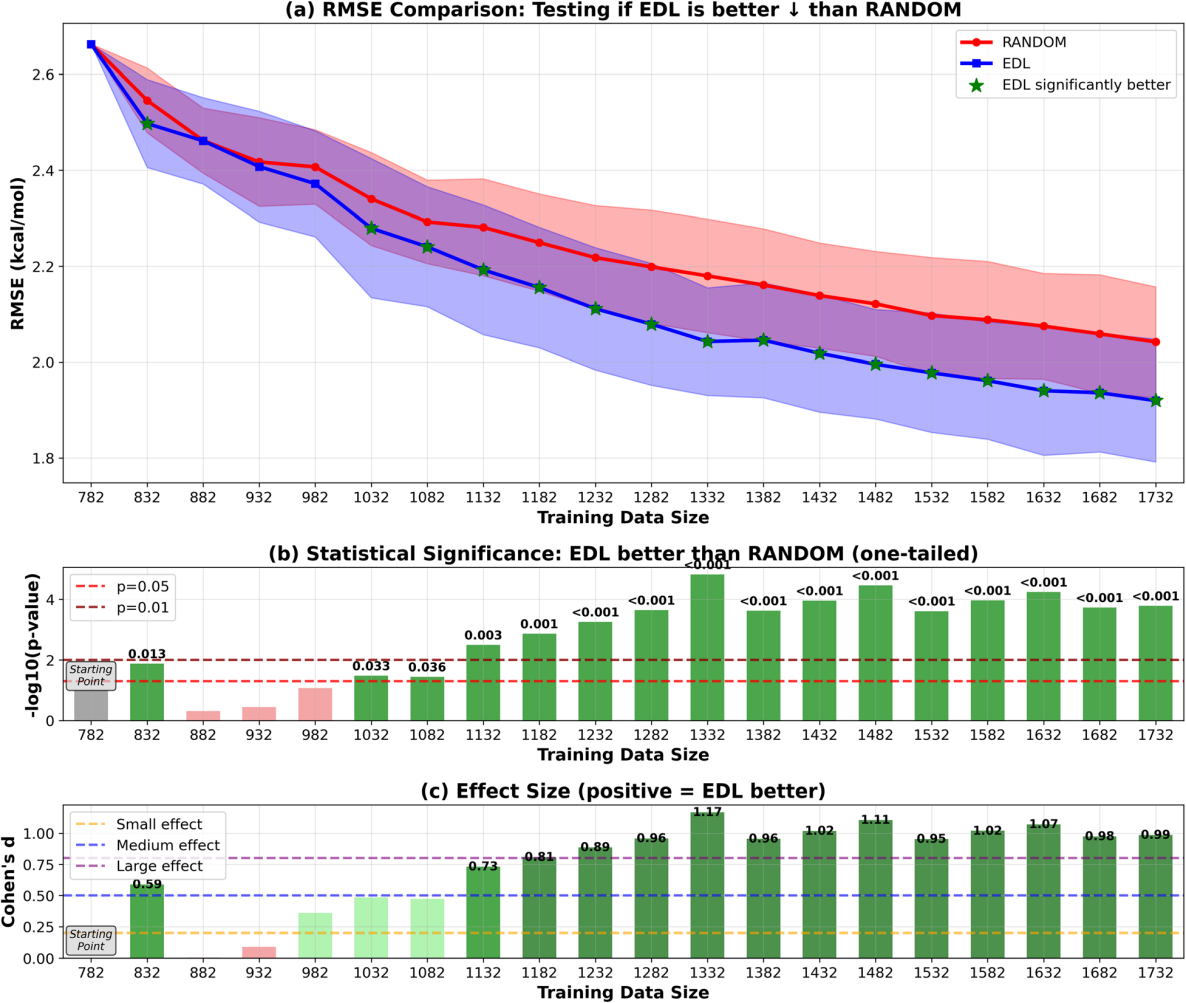
RMSE results of EDL compared with random sampling, starting with a matched-size random subset from SOMAS and generalizing to the PubChem dataset. Each iteration adds 50 samples. Top panel (a) shows mean ± standard deviation for RMSE, with green stars indicating statistically significant improvements (*p* < 0.05). The arrow in the panel title indicates the direction of improvement for the plotted metric. ↓ denotes that lower values indicate better performance (RMSE), whereas ↑ denotes that higher values indicate better performance (*R*^2^). Middle panel (b) displays statistical significance levels (−log_10_ *p*-values) from one-tailed tests, with horizontal dashed lines at *p* = 0.05 and *p* = 0.01 thresholds. Bottom panel (c) shows Cohen's d effect sizes, with horizontal lines indicating small (0.2), medium (0.5), and large (0.8) effect thresholds. Green coloring indicates significant improvements with meaningful effect sizes.

**Fig. 10:**
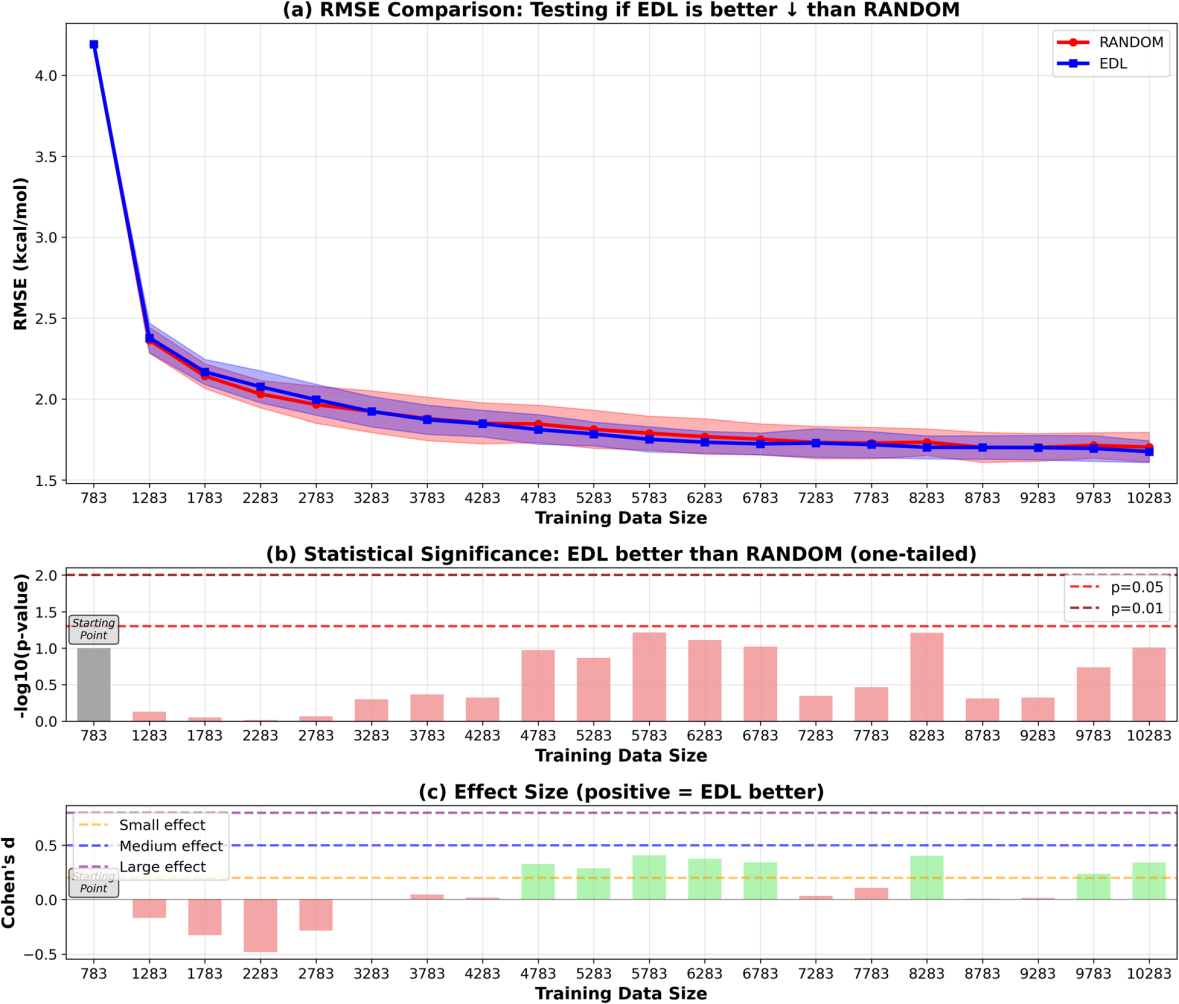
RMSE results of EDL compared with random sampling, starting with the CHO subset from SOMAS and generalizing to the PubChem dataset. Each iteration adds 500 samples. Top panel (a) shows mean ± standard deviation for RMSE, with green stars indicating statistically significant improvements (*p* < 0.05). The arrow in the panel title indicates the direction of improvement for the plotted metric. ↓ denotes that lower values indicate better performance (RMSE), whereas ↑ denotes that higher values indicate better performance (*R*^2^). Middle panel (b) displays statistical significance levels (−log_10_ *p*-values) from one-tailed tests, with horizontal dashed lines at *p* = 0.05 and *p* = 0.01 thresholds. Bottom panel (c) shows Cohen's d effect sizes, with horizontal lines indicating small (0.2), medium (0.5), and large (0.8) effect thresholds. Green coloring indicates significant improvements with meaningful effect sizes.

**Fig. 11:**
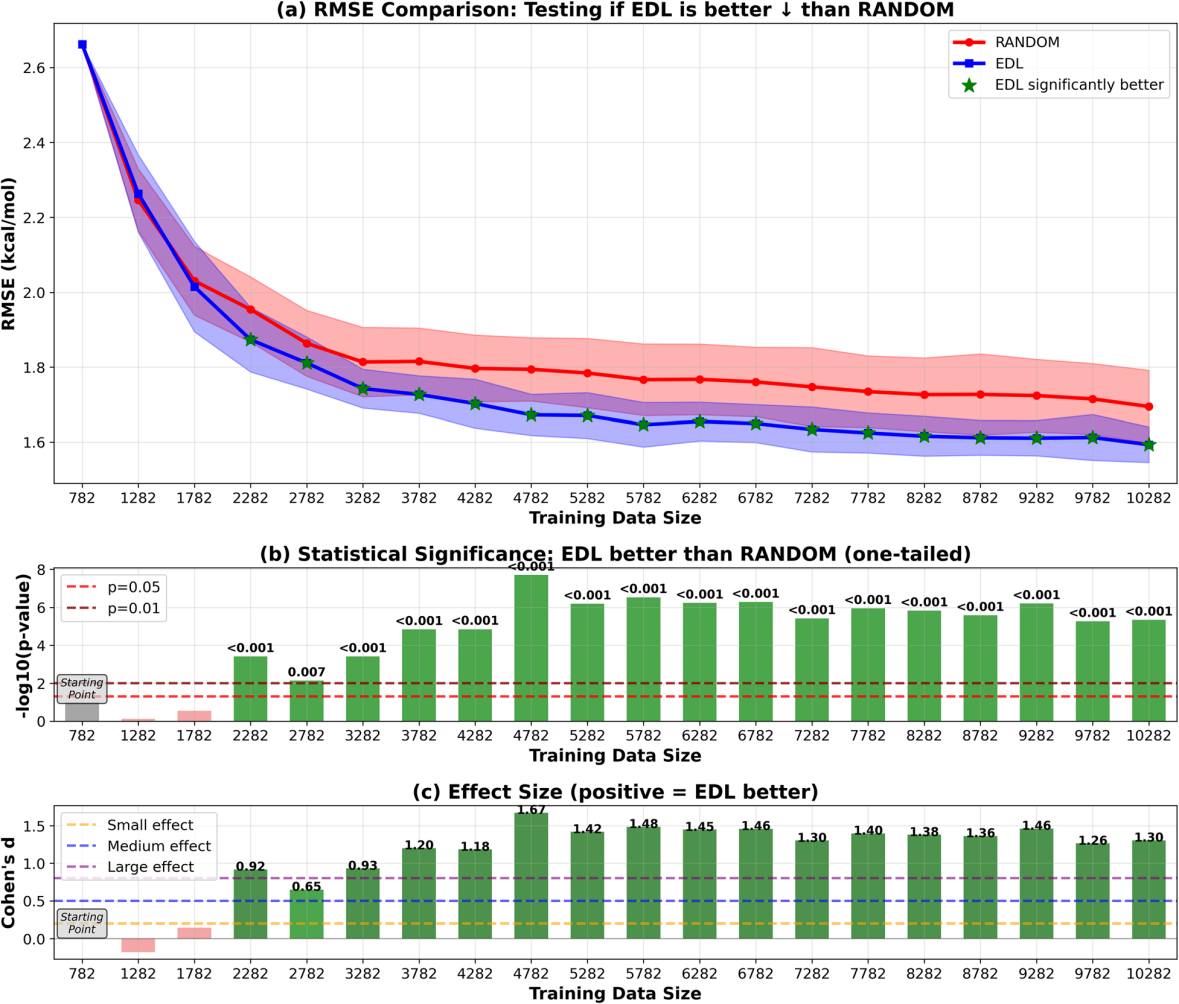
RMSE results of EDL compared with random sampling, starting with a matched-size random subset from SOMAS and generalizing to the PubChem dataset. Each iteration adds 500 samples. Top panel (a) shows mean ± standard deviation for RMSE, with green stars indicating statistically significant improvements (*p* < 0.05). The arrow in the panel title indicates the direction of improvement for the plotted metric. ↓ denotes that lower values indicate better performance (RMSE), whereas ↑ denotes that higher values indicate better performance (*R*^2^). Middle panel (b) displays statistical significance levels (−log_10_ *p*-values) from one-tailed tests, with horizontal dashed lines at *p* = 0.05 and *p* = 0.01 thresholds. Bottom panel (c) shows Cohen's d effect sizes, with horizontal lines indicating small (0.2), medium (0.5), and large (0.8) effect thresholds. Green coloring indicates significant improvements with meaningful effect sizes.

Because the CHO subset is both much smaller and less diverse than the full SOMAS data, we aim to disentangle these two factors by initiating the active learning framework with a randomly selected subset of comparable size. The result is shown in Fig. 9 for the 50-sample experiment. In this scenario, active learning demonstrated a bigger advantage over random sampling with a consistent AL advantage of 0.1 kcal mol^−1^. The observed difference can be attributed to the initial dataset's diversity: when commencing with a specific subset of reduced diversity, the uncertainty quantification model's performance in estimating uncertainty is suboptimal, leading to less effective selection of unknown molecules. The results of the 500-sample experiments for CHO and random subsets are shown in Fig. 10 and 11.

**Fig. 12:**
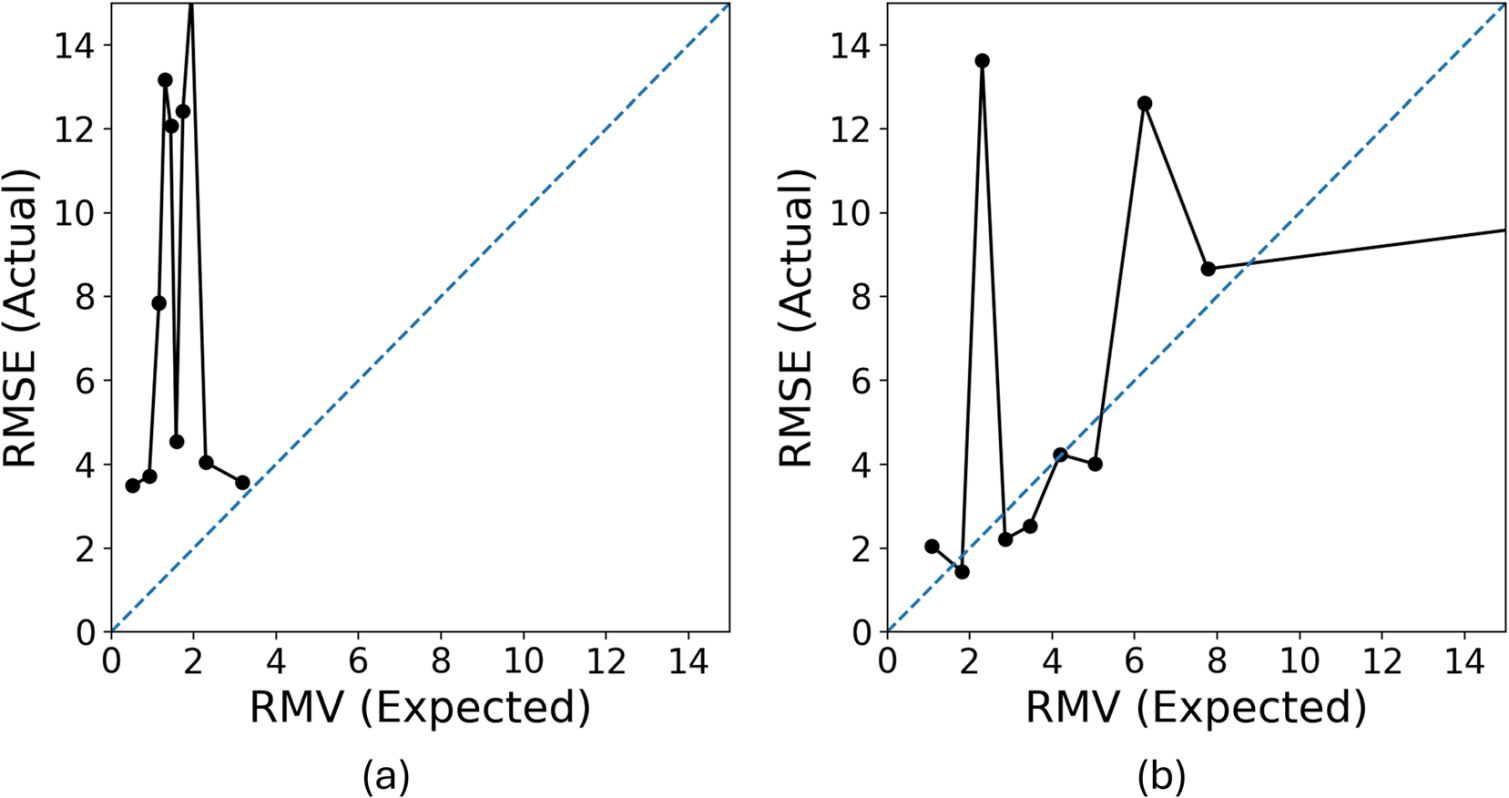
(a) The calibration plot of the model trained with the CHO subset. The calibration plot illustrates the uncertainty quantification performance. The *x*-axis represents the square root of the mean variance RMV (expected error from uncertainty estimates), and the *y*-axis shows the actual root mean square error (RMSE). The data is grouped into 10 bins for evaluation, with perfect calibration aligned along the diagonal. (b) The calibration plot of the model trained with the matched-size subset.

To validate this hypothesis, we evaluated the uncertainty quantification performance of models trained on either the CHO subset or the random subset of the same size, using a test set of approximately 1000 molecules from the PubChem pool. The Expected Normalized Calibration Error (ENCE) values,^20^ presented in Table 1, indicate that the model trained on the random subset achieved satisfactory ENCE, while the CHO-trained model exhibited poor ENCE. Furthermore, the calibration plots in Fig. 12 corroborate these findings: although not perfect, the model trained on the random subset demonstrates better calibration compared to the CHO-trained model.

**Table 1:** ENCEs of the models trained with either the CHO subset or the random subset

Start subset	ENCE
CHO	4.67
Random	0.86

In the 500-sample experiment, the CHO subset does not exhibit any significant difference between EDL and random sampling, whereas the random subset still demonstrates a 0.1 kcal mol^−1^ advantage. This behavior may be attributed to the CHO subset being unrepresentative, which limits its effectiveness for uncertainty quantification. While the CHO subset still provides some value, evidenced by a slight improvement over random when adding samples incrementally (*e.g.*, 50 at a time), its advantage diminishes when adding a larger number of samples (*e.g.*, 500 at a time), resulting in performance comparable to random sampling. Importantly, the results for the random subset in the 500-sample experiment align with the findings in Fig. 5, where EDL consistently outperforms random sampling until the dataset size approaches approximately 10 000 samples.

## 3. Discussion and conclusions

The primary goal of this study was to evaluate the effectiveness of AL frameworks, particularly EDL, in enhancing molecular property prediction. While AL has shown potential in numerous applications, the specific challenge of true OOD generalization, which is critical for real-world scenarios, has often remained insufficiently addressed in previous studies. By systematically analyzing EDL-based AL, we aimed to provide a deeper understanding of its advantages over random sampling and its potential limitations.

Our findings demonstrate that EDL-based AL consistently offers advantages over random sampling in molecular property prediction tasks. For the in-SOMAS experiment, EDL provided an improvement of approximately 0.05 kcal mol^−1^. This advantage was also maintained when generalizing to the PubChem dataset, where EDL achieved a similar 0.05 kcal mol^−1^ improvement. These results highlight the ability of EDL to consistently enhance prediction performance compared to random sampling strategies across datasets.

A critical insight emerging from our analysis is that the success of AL does not come from simply matching the distribution of the training set to the testing set. While random sampling achieves higher similarity between the training and hold-out sets, as quantified by FCD, EDL-based AL instead prioritizes the inclusion of more informative and diverse molecules into the training set. This targeted inclusion improves the model's capacity to generalize to unseen data, as opposed to focusing solely on common types of molecules emphasized by random sampling.

Our data also revealed nuanced behavior when starting from different subsets of the initial training set. When beginning with a random subset, for the 50-sample experiment, EDL maintained an advantage over random sampling, improving RMSE by 0.1 kcal mol^−1^. This advantage was reduced to 0.05 kcal mol^−1^ when starting from the biased CHO subset, due to the CHO subset being less representative of the hold-out distribution. Similarly, in the 500-sample experiment, the CHO subset failed to demonstrate a significant difference between EDL and random sampling, while the random subset maintained a 0.1 kcal mol^−1^ advantage. This can be attributed to the biased nature of the CHO subset, which hinders the effectiveness of uncertainty estimation and thereby limits the informed selection of new samples during AL. Thus, our results suggest that a more diverse and representative initial training set is crucial for optimizing the benefits of AL.

While EDL yields an average RMSE improvement of approximately 0.05 kcal mol^−1^ over random sampling, we acknowledge that this effect size is modest. Accordingly, our contribution is not to claim a new level of accuracy gain, but to provide controlled evidence that EDL acquisition can produce consistent improvements over random selection in an OOD AL setting. Evaluating the approach on additional molecular properties is an important direction for future work and may reveal larger benefits.

In conclusion, our study supports the effectiveness of EDL-based AL in molecular property prediction, with consistent advantages over random sampling observed in both in-distribution and OOD generalization tasks. The success of AL arises from its ability to selectively include diverse and informative molecules, rather than relying solely on distribution-matching with the hold-out dataset. However, our analysis also highlights the importance of initializing AL with a diverse and representative dataset, as biased or overly limited subsets can severely hinder its performance. Future studies should explore strategies for improving initialization, such as hybrid approaches that combine random and informed sampling, evaluating the approach on new molecular properties, and focus on further enhancing uncertainty quantification in OOD scenarios.

## 4. Methodology

### 4.1 Datasets

The Solubility of Organic Molecules in Aqueous Solution (SOMAS) dataset^17^ containing about 12 000 molecules that covers wider chemical and solubility regimes. In this study we filter the SOMAS and PubChem datasets to remove several types of molecules that require excessive solvation free energy calculation time. The molecules that are composed of more than 50 atoms (hydrogen atoms are not counted) were removed. Additionally, all ions are not included due to the larger error in the solvation free energy calculation.^21,22^ PubChem^18^ is a comprehensive and publicly accessible database maintained by the National Center for Biotechnology Information (NCBI) at the National Institutes of Health (NIH). In our study, we randomly selected approximately 40 000 compounds from the PubChem database to evaluate the generalization capability of our active learning approaches. We perform the same filtering of molecules from the PubChem dataset leading to 22 573 molecules. Table 2 shows the dataset sizes.

**Table 2:** Sizes of the datasets

Dataset	Size
SOMAS	8795
SOMAS(CHO)	869
PubChem	22 573

In assessing the performance of active learning, dataset diversity plays a crucial role. Table 3 presents the entropy estimator, as described by Leguy *et al.* (2021),^23^ for various datasets under consideration. The high entropy values observed across our datasets indicate diversity, which enhances the generalizability of our findings.

**Table 3:** Diversity of the datasets by entropy estimator

	Scaffold entropy	IFG entropy	Shingles entropy	Mean
SOMAS	7.76	4.48	3.99	5.41
SOMAS(CHO)	6.17	2.67	2.83	3.89
PubChem	9.52	4.59	4.06	6.06

### 4.2 Solvation free energy calculation

Solvation free energy is a fundamental thermodynamic property that quantifies the energy change when a solute is transferred from vacuum (or gas phase) into a solvent. This property is crucial for understanding molecular behavior in solution, as it governs solubility, partition coefficients, and the driving forces behind molecular recognition and self-assembly processes. Accurate prediction of solvation free energies is essential for applications ranging from drug design and life science to materials science. In this work, the solvation free energy was calculated using Vyboishchikov and Voityuk's method. It was represented by the sum of the electrostatic energy *E*_elst_ and the correction term.Δ*G*_solv_ = *E*_elst_ + Δ*G*^0^_corrsolv_

For the details, see the papers by Vyboishchikov and Voityuk.^21,22,24^

**Fig. 13:**
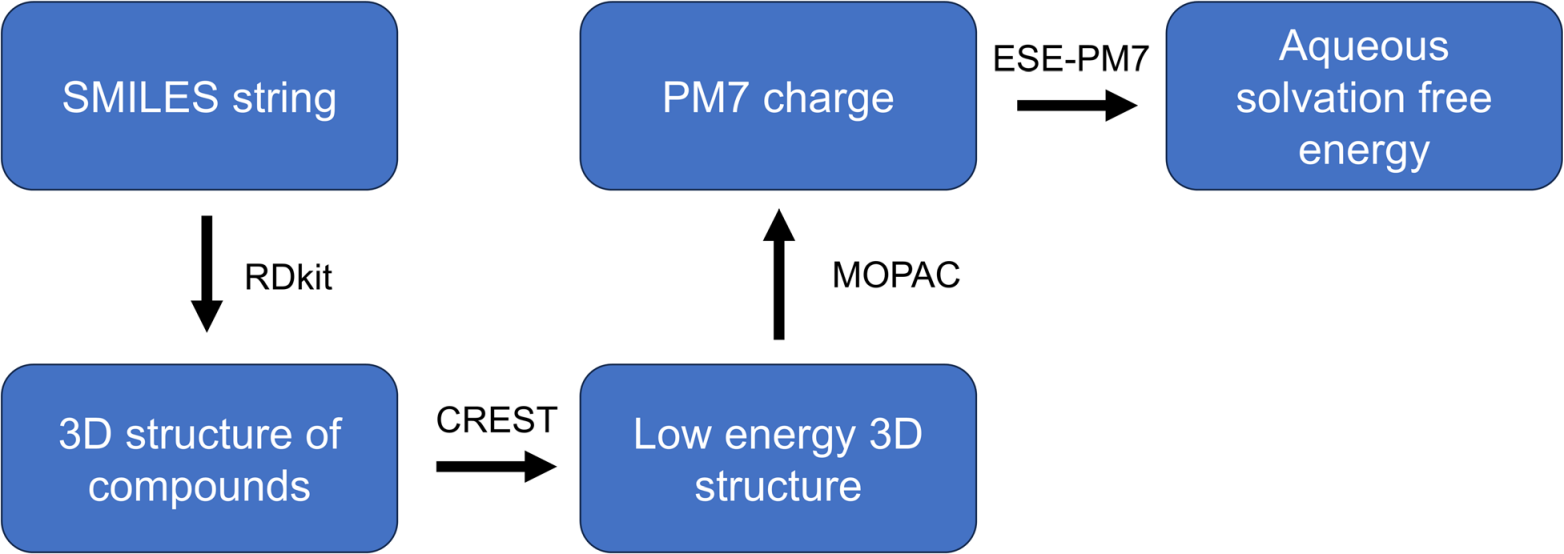
Flowchart of calculating the solvation energy.

Fig. 13 shows the workflow for the solvation free energy calculation. The Simplified molecular-input line-entry system (SMILES) string input of the molecules is converted to 3D chemical structure with RDKit (https://www.rdkit.org/).^25^ Conformer–Rotamer Ensemble Sampling Tool (CREST)^26^ was used to generate the conformers and search the low energy conformers of the molecule with the GFN2-xTB method,^27^ the analytical linearized Poisson–Boltzmann (ALPB) solvation model and the default workflow. The low energy conformer was further optimized by MOPAC with PM7 method^28^ (http://openmopac.net)^29^ and then the partial charges were calculated. The ESE-PM7 software^21^ was employed to calculate the aqueous solvation free energy of the molecule using the atomic charges as input.

### 4.3 Active learning framework

The active learning framework is designed to iteratively improve a model's performance by selectively adding informative samples to the training set. Initially, the model is trained on an initial training set. At each iteration, new molecules are selected based on their potential to improve the model, using strategies such as uncertainty estimation from EDL (see Section 4.4) or random selection. The solvation free energy of these new molecules are calculated and then added to the training set, and the model is retrained using the new training set. This process continues until a predefined stopping criterion is met, such as a maximum number of iterations.

In this specific implementation, the framework can be summarized as:

(1) Initializes with data from the SOMAS dataset.

(2) At each step, adds N molecules from the PubChem dataset, chosen either randomly or based on their uncertainty estimates. Calculate their solvation free energy.

(3) Retrains the model with the augmented training set.

(4) Evaluates the model performance on a hold-out test set using RMSE and *R*^2^.

(5) Compares the performance of EDL and random selection strategies.

More details are in Algorithm 1.
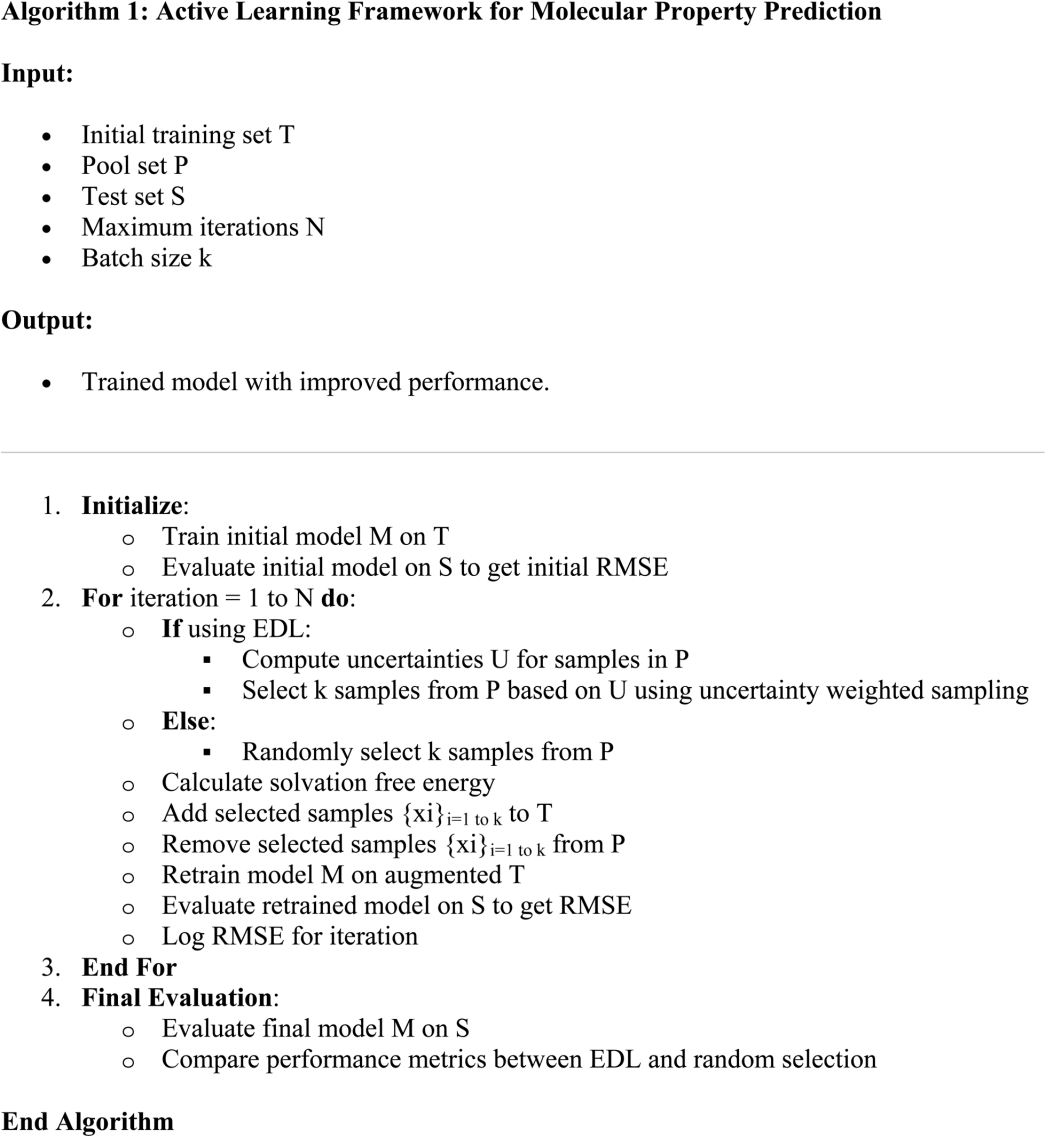


### 4.4 EDL and model training

EDL is an uncertainty quantification method for deep learning models that aims to predict the parameters of a distribution of the predictive likelihood function for each prediction rather than simply predicting the target value. The method applies a prior distribution on the likelihood parameters *µ*(*x*) and *σ*^2^(*x*) and approximates the posterior distribution of these parameters using a Normal Inverse-Gamma (NIG) distribution.^6^ The predicted values of the variance *σ*^2^(*x*) are used as the measure of model uncertainty for each prediction. We implement EDL using the molecular descriptor model (MDM) and the features derived from the molecular structure^30^ as inputs to the model.

The deep learning architecture of our model consists of a series of dense layers. Initially, the input layer is connected to a dense layer followed by a dropout layer to prevent overfitting. This structure is repeated for the second dense layer, followed by another dropout layer. The final layer is a custom layer designed for evidential regression, which outputs the parameters for a normal gamma distribution. The model is compiled with the Adam optimizer and a custom loss function designed for evidential regression. Early stopping is employed, monitoring the validation loss with a patience, to prevent overfitting and ensure efficient training. In each experiment, we use 10% of the training data as the validation set during model training to evaluate the model's performance on unseen data, ensuring the model generalizes well (Table 4).

**Table 4:** Hyper-parameters for the deep learning model

Hyper-parameter	Value
Dense layer dimensions	(128, 576)
Dropout rates	(0.11, 0.60)
Learning rate	0.0005
Batch size	64
Epochs	1000
Early stop patience	25
Validation split	0.1

The entire AL process can take hours, often extending to 2–3 days on cluster, which depends on the size of the dataset. The most time-consuming step is the calculation of the SFE. In contrast, the training of the deep learning model with EDL is relatively quick, typically taking less than a minute. However, the data generation part, *i.e.*, the SFE calculation, particularly the low energy configuration search by CREST,^26^ significantly contributes to the overall time. The typical time for low energy configuration search is 0.5 to 3 hours on an AMD EPYC 7502 CPU with 64 cores. The time of other parts in the SFE calculation flow is just a couple of minutes. While the data generation process can be independent when the molecule dataset is fixed, the active learning component can directly access and read the data.

### 4.5 Frechet ChemNet distance

The Fréchet ChemNet Distance (FCD)^19^ is a metric designed to measure the similarity between distributions of chemical representations generated by neural networks. This metric draws inspiration from the Fréchet Inception Distance (FID)^31^ used in evaluating generative models in computer vision. The FCD assesses the performance of generative models in the field of chemistry by comparing the distributions of latent representations of generated molecules against those of real molecules. These representations are typically obtained using a pre-trained neural network, such as ChemNet.^19^ To compute the FCD, the generated and real molecules are first passed through the ChemNet to obtain their respective latent representations. The mean and covariance of these representations are then calculated for both sets of molecules. The Fréchet distance between these two distributions is subsequently determined, with a lower FCD indicating a higher similarity between the generated molecules and the real ones.

### 4.6 Evaluation metrics and statistical testing

RMSE and *R*^2^ are two widely used metrics for evaluating the performance of regression models. RMSE measures the average difference between the estimated values and the actual values, providing a clear indicator of the model's prediction accuracy. On the other hand, *R*-squared is a statistical measure that represents the proportion of the variance for a dependent variable that's explained by the independent variables in the model, which ranges from 0 to 1.

We evaluate predictive performance primarily using RMSE on a held-out test set, as it reflects the typical magnitude of prediction error. Because AL is stochastic, we repeat each experiment across 30 independent runs and report aggregate statistics. To quantify whether observed differences between strategies are robust across runs, we use two complementary statistics:

(1) *p*-Value (hypothesis testing). For each EDL *vs.* random comparison, we compute a *p*-value to test the null hypothesis that there is no difference in mean performance across runs. A smaller *p*-value indicates that the observed difference is unlikely to be explained by run-to-run variability alone. We emphasize that *p*-values assess statistical evidence, not the magnitude of an improvement.

(2) Standardized effect size (Cohen's d). We additionally report Cohen's d to measure the magnitude of the difference between two methods. Cohen's d is computed as the difference in group means divided by the pooled standard deviation; larger absolute values indicate stronger separation between methods. In our figures, the sign of d reflects which method performs better, while its magnitude summarizes practical relevance relative to run-to-run variation.

Together, *p*-values and Cohen's d provide a more complete picture than mean RMSE alone.

## Author contributions

ES conceived the idea and directed the research. TY performed the formal analysis and software development. PG conducted the solvation free energy calculation part. GP provided the PubChem dataset. TY wrote the original draft while ES reviewed and edited.

## Conflicts of interest

All authors declare no financial or non-financial competing interests.

## Supplementary Material

RA-016-D5RA08055J-s001

## Data Availability

The datasets generated and/or analysed during the current study are available at https://zenodo.org/records/13769710. Code availability: the active learning loop code is available at https://github.com/pnnl/UQALE/tree/master/OOD_active_learning. Supplementary information: the *R*^2^ results of the deep learning model evaluations on the test sets. See DOI: https://doi.org/10.1039/d5ra08055j.
