# Supplementary material for: Out-of-distribution evaluation of active learning pipelines for molecular property prediction
Source: RSC Adv. 2026 Jan 23;16(6):5281–95. doi: 10.1039/d5ra08055j (PMC12829442; doi:10.1039/d5ra08055j)
Supplement: RA-016-D5RA08055J-s001 [file RA-016-D5RA08055J-s001.pdf]

This journal is © The Royal Society of Chemistry 2026

Appendix

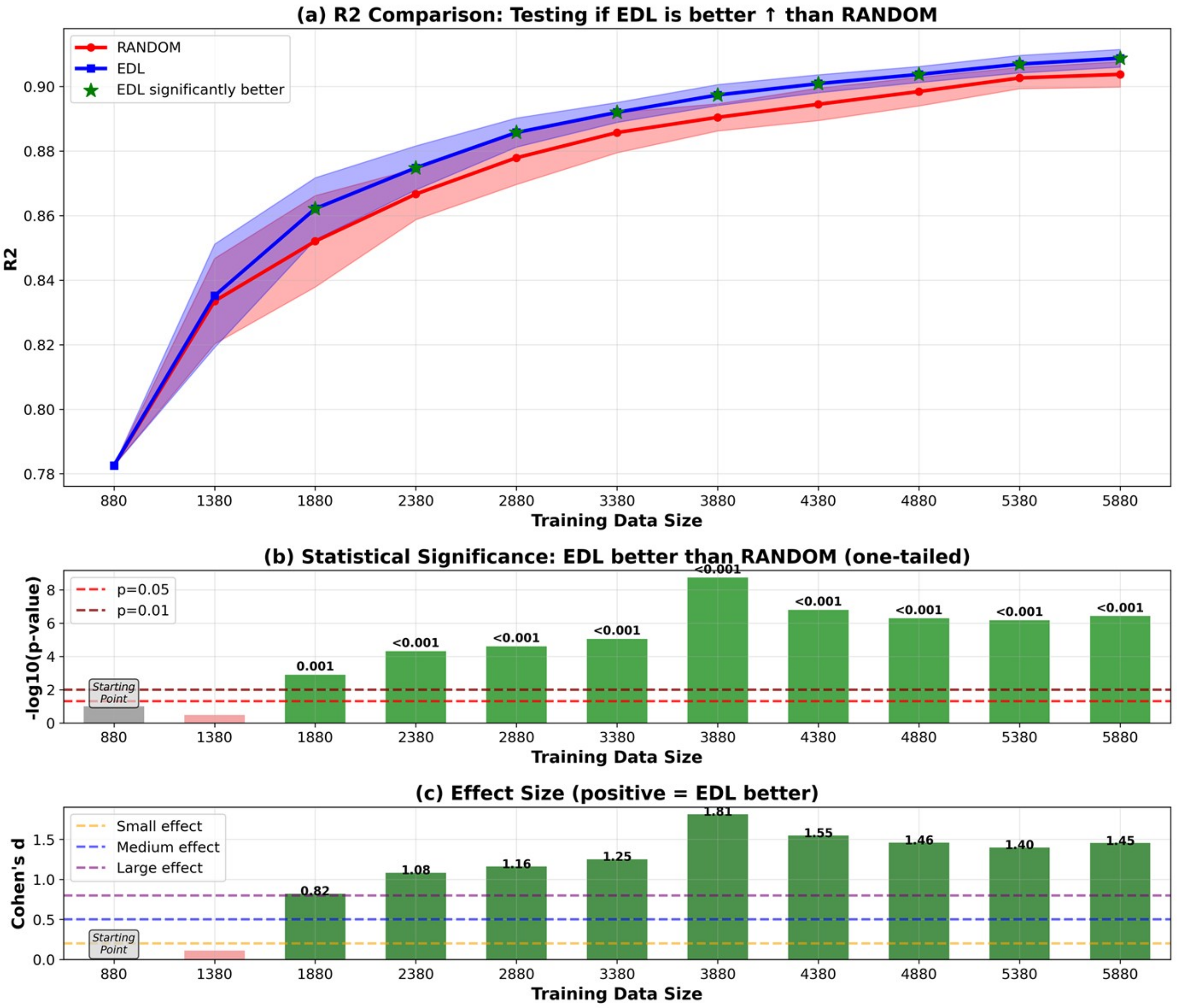

Figure A1. R^2 results of EDL compared with random sampling within the SOMAS dataset. Each iteration adds 500 samples.

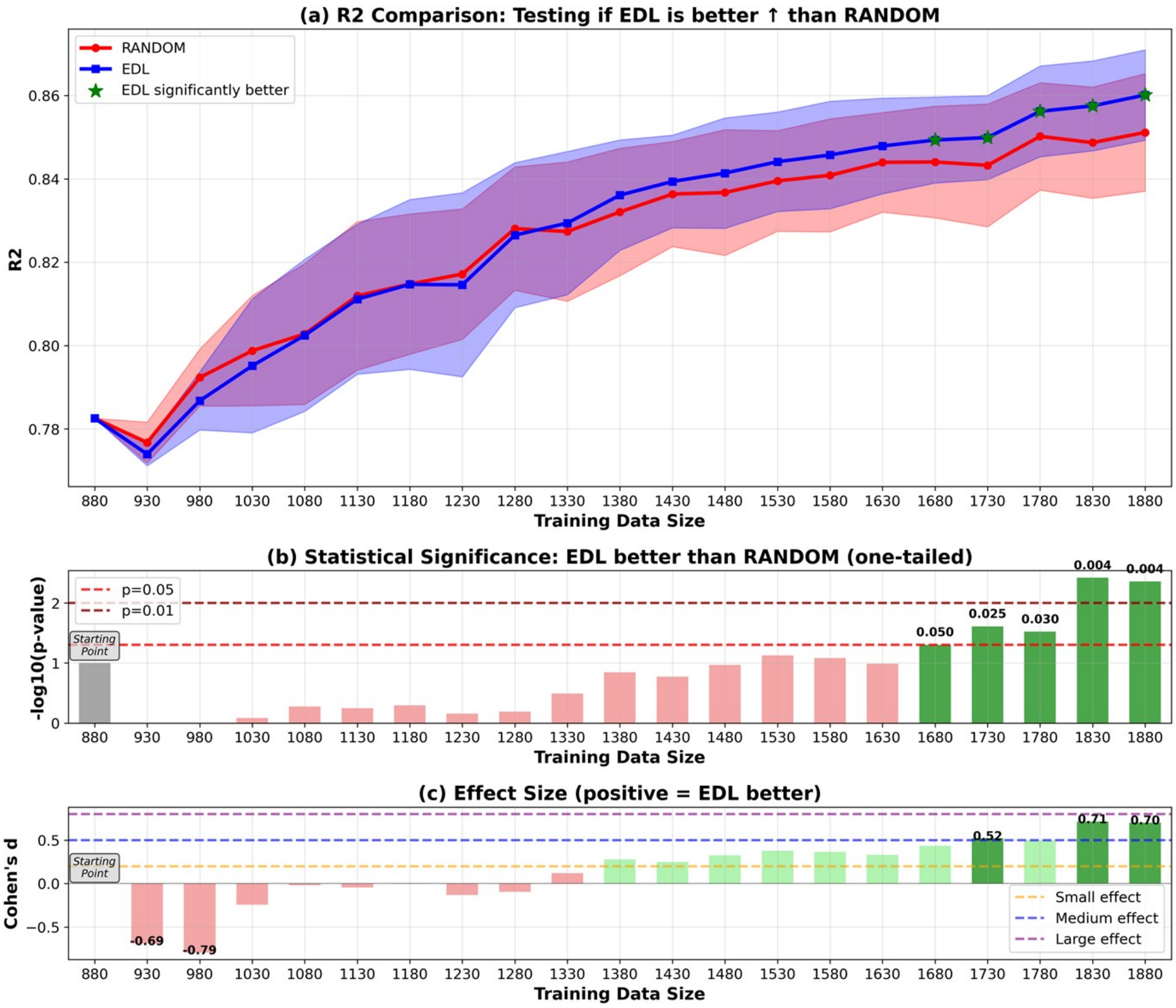

Figure A2. R^2 results of EDL compared with random sampling within the SOMAS dataset. Each step adding 50 samples.

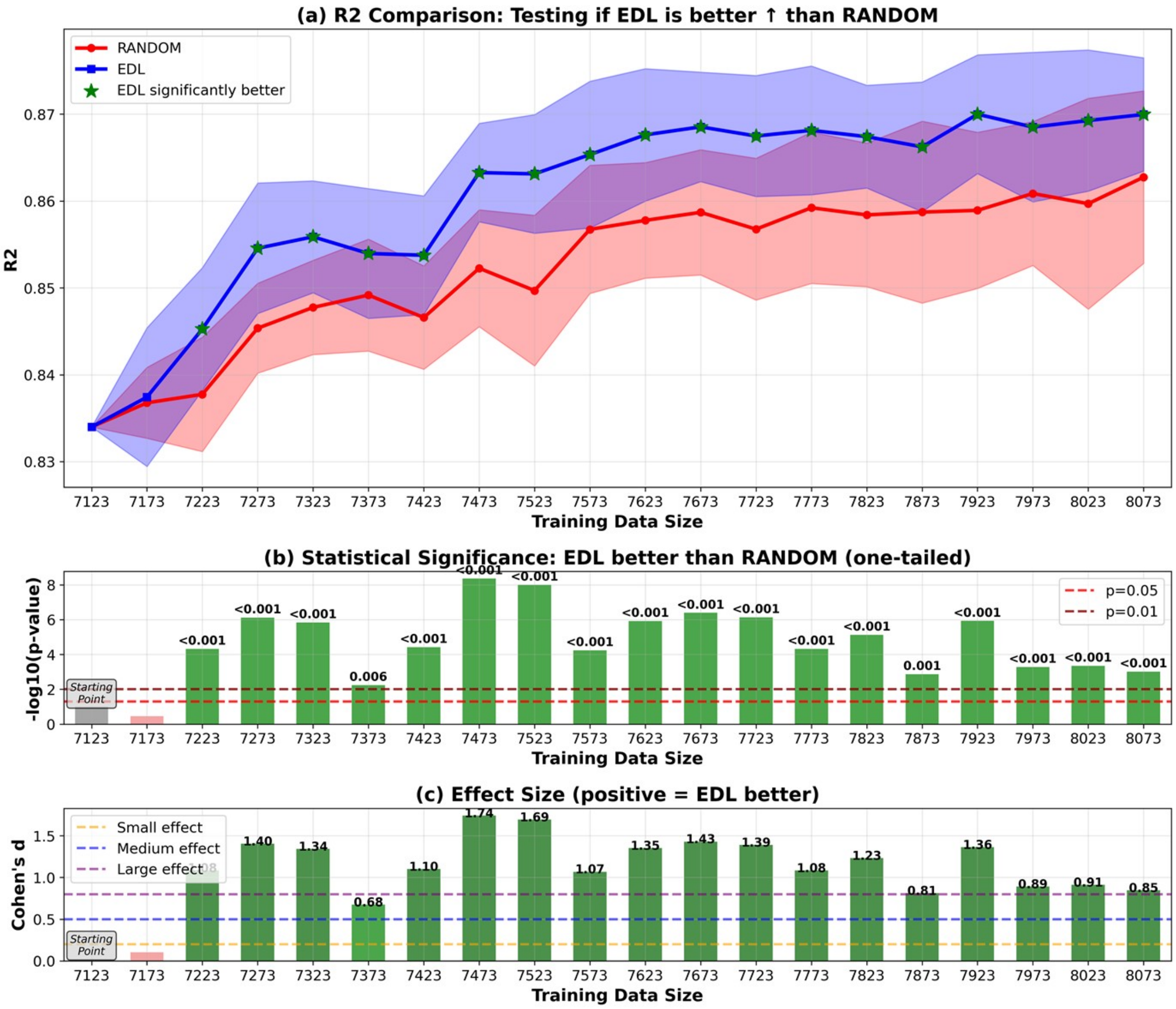

Figure A3. R^2 results of EDL compared with random sampling, starting with the SOMAS dataset and generalizing to the PubChem dataset. Each iteration adds 50 samples.

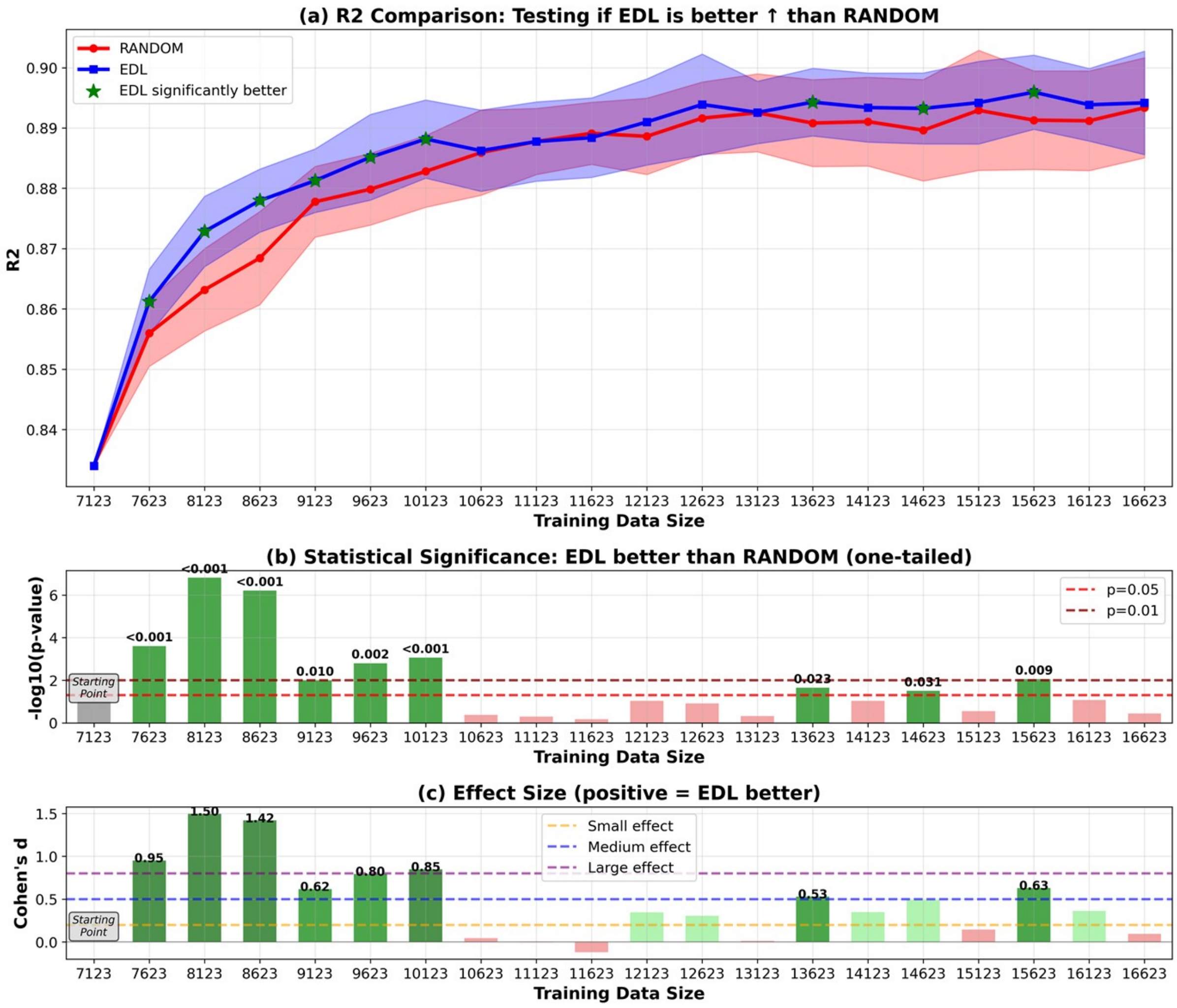

Figure A4. R^2 results of EDL compared with random sampling, starting with the SOMAS dataset and generalizing to the PubChem dataset. Each iteration adds 500 samples.

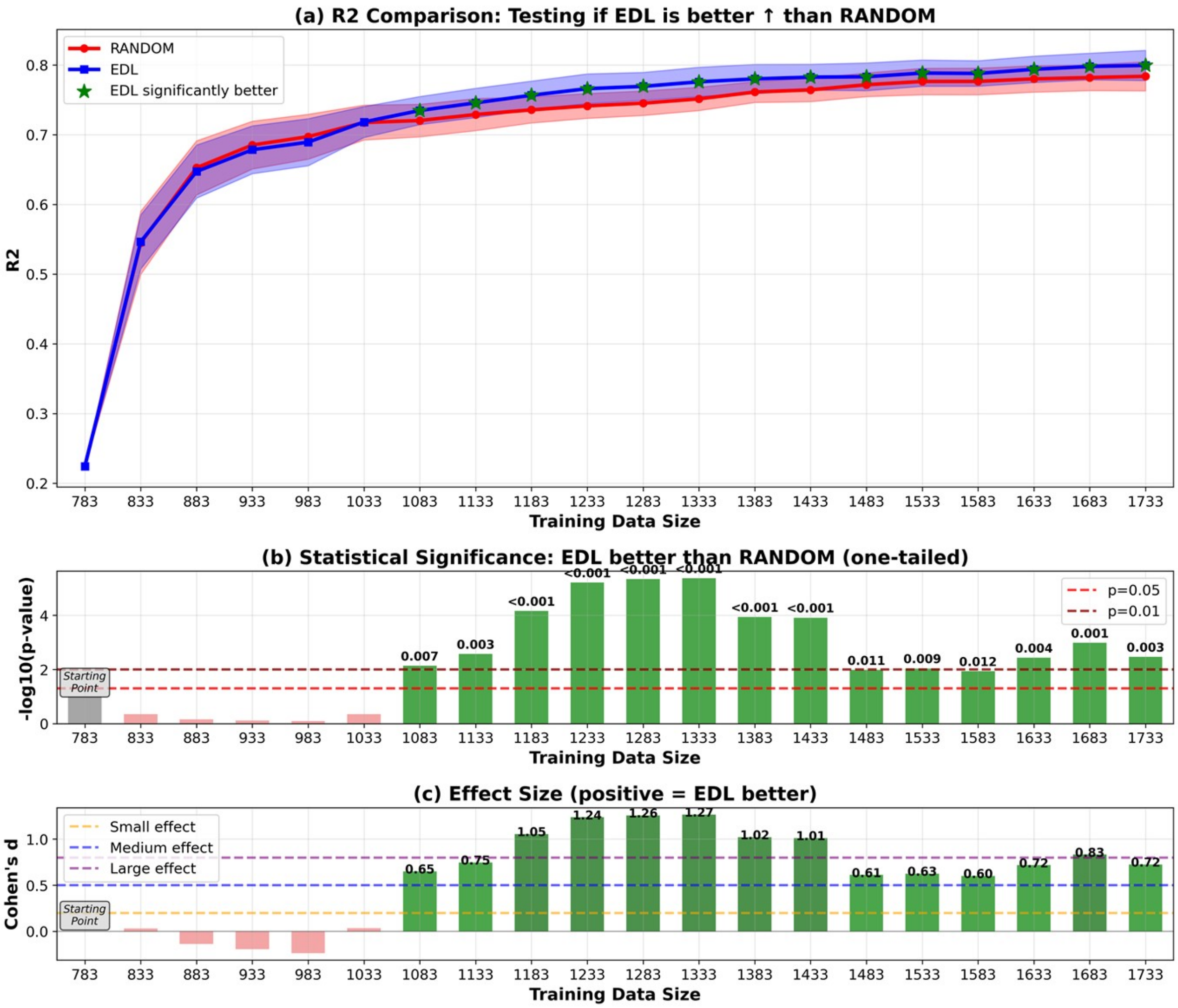

Figure A5. R^2 results of EDL compared with random sampling, starting with the CHO subset from SOMAS and generalizing to the PubChem dataset. Each step adding 50 samples.

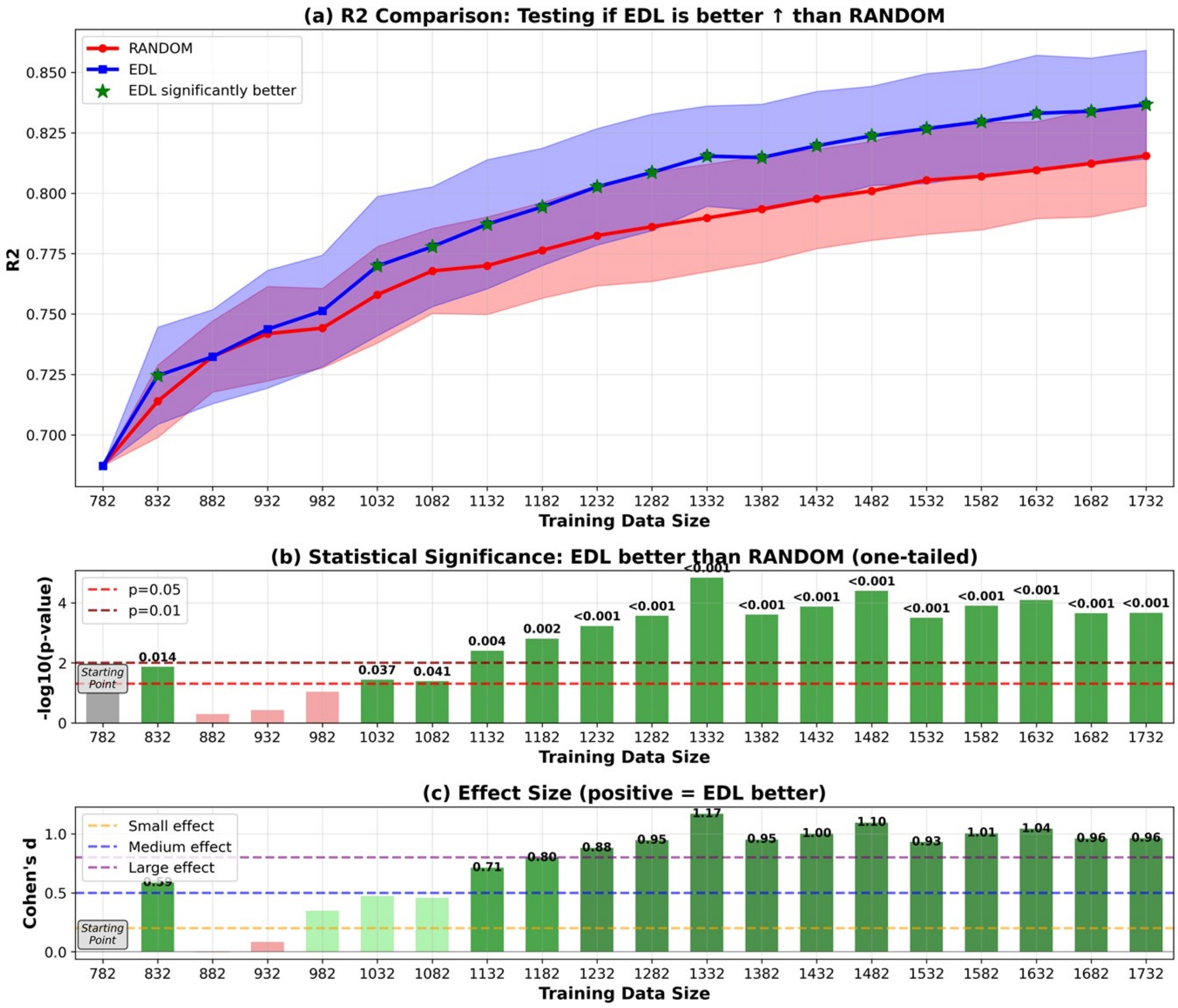

Figure A6. R^2 results of EDL compared with random sampling, starting with a matched-size random subset from SOMAS and generalizing to the PubChem dataset. Each iteration adds 50 samples.

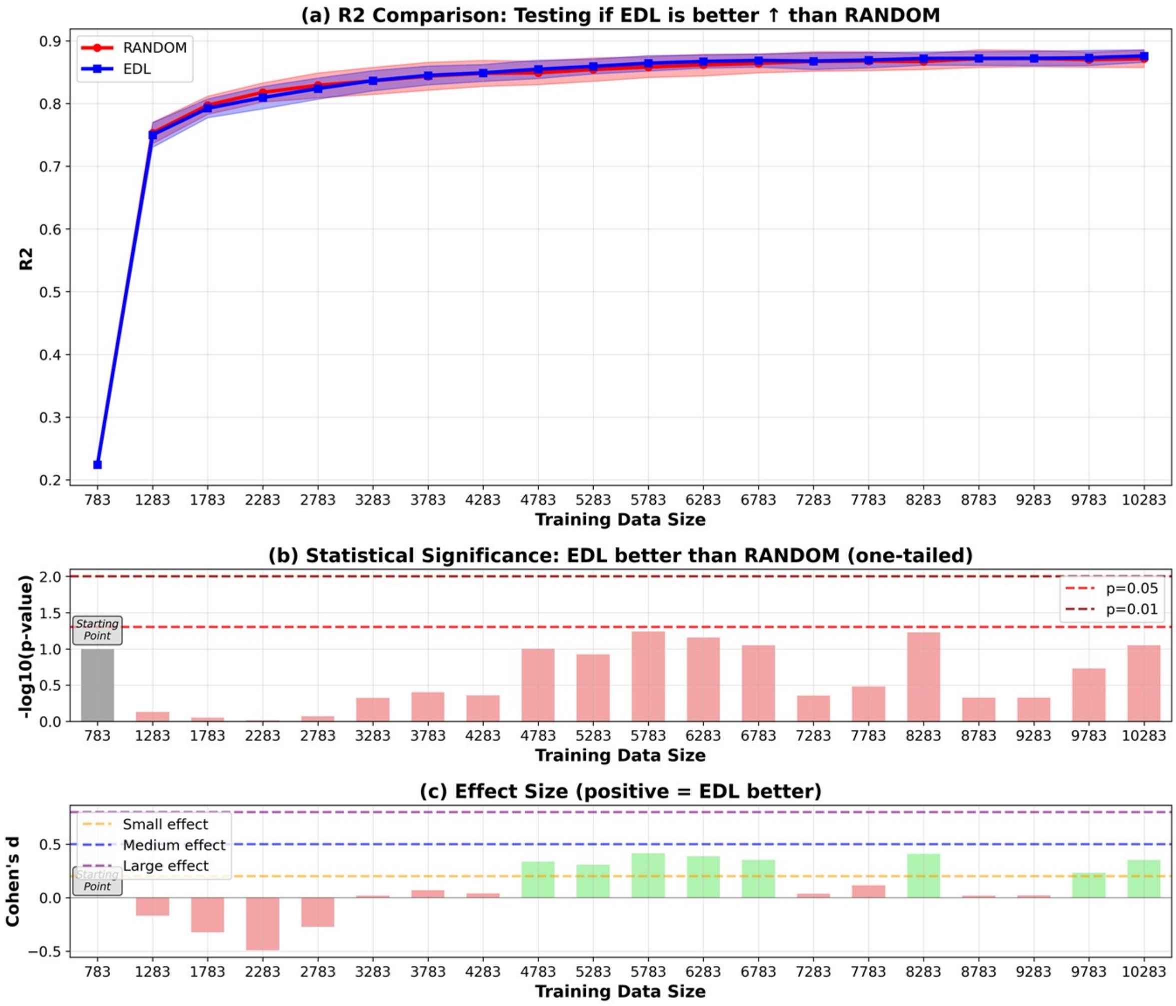

Figure A7. R^2 results of EDL compared with random sampling, starting with the CHO subset from SOMAS and generalizing to the PubChem dataset. Each iteration adds 500 samples.

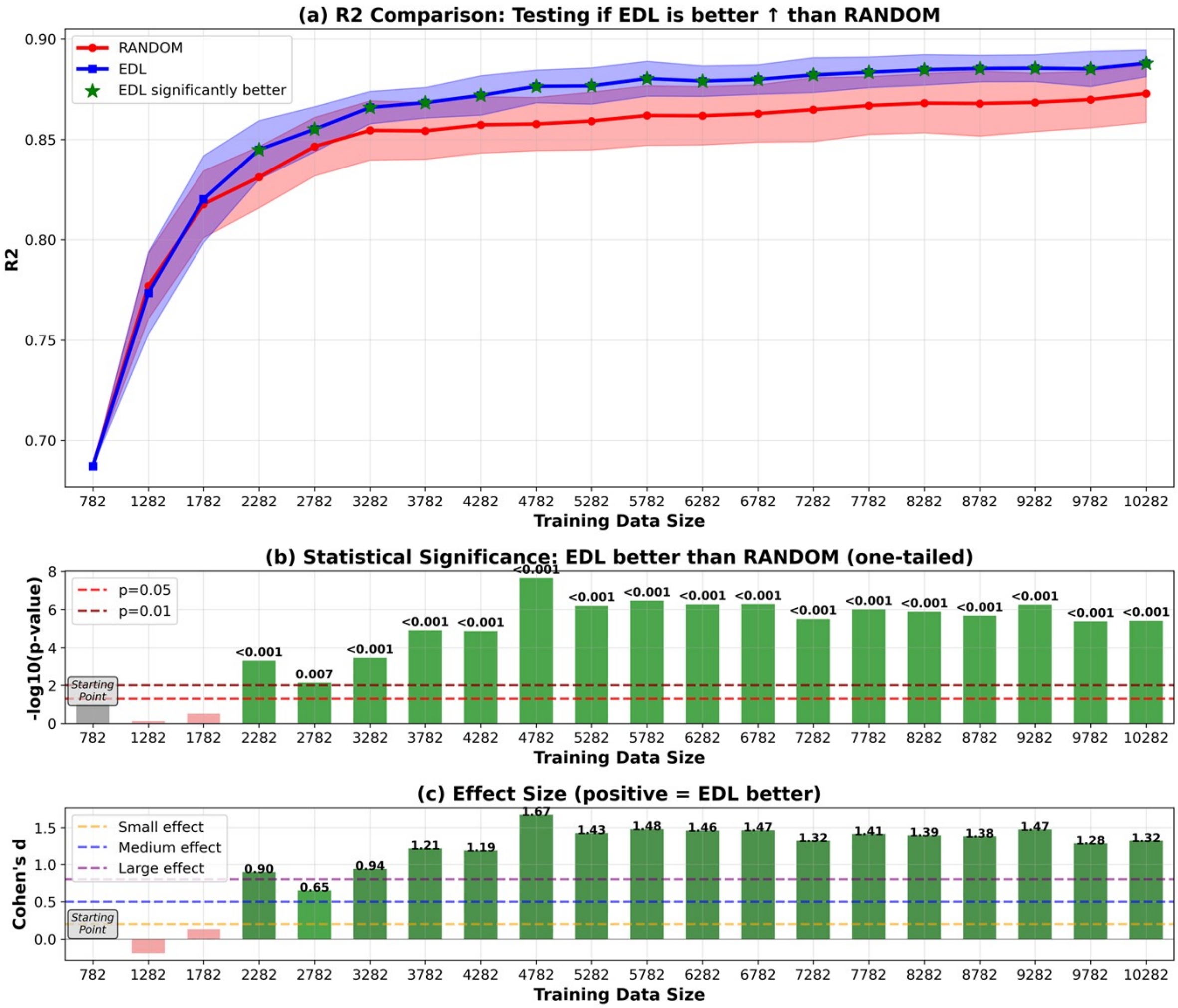

Figure A8. R^2 results of EDL compared with random sampling, starting with a matched-size random subset from SOMAS and generalizing to the PubChem dataset. Each iteration adds 500 samples.
